# Assessment of the Physicochemical, Textural, Sensory, and Antioxidant Properties of Sorbet Enriched with Alfalfa Protein Concentrate

**DOI:** 10.3390/foods15193504

**Published:** 2026-09-30

**Authors:** Viorica Bulgaru, Angela Gurev, Iana Ciugureanu, Sorina Ropciuc, Aliona Ghendov-Mosanu

**Affiliations:** 1 Faculty of Food Technology, Technical University of Moldova, 9/9 Studentilor St., MD-2045 Chisinau, Moldova; viorica.bulgaru@tpa.utm.md (V.B.); angela.gurev@chim.utm.md (A.G.); iana.ciugureanu@doctorat.utm.md (I.C.); 2 Faculty of Food Engineering, “Stefan cel Mare” University of Suceava, 13 University St., 720229 Suceava, Romania; sorina.ropciuc@fia.usv.ro

**Keywords:** alfalfa protein concentrate, apple sorbet, bioactive compounds, functional food, plant-based protein

## Abstract

This study investigated alfalfa protein concentrate (APC) as a functional ingredient for developing protein-enriched apple sorbet. APC obtained by ultrasound-assisted aqueous extraction was characterized for its physicochemical, nutritional, phytochemical, antioxidant, textural, and functional properties and incorporated into sorbet at 2.5–10%. APC contained 86.45% protein (DW), 2297.4 mg GAE/100 g DW total polyphenols, and 983.6 mg QE/100 g DW total flavonoids. APC incorporation increased sorbet protein content from 0.11% in the control sample to 8.05% at 10% APC, while total polyphenols increased from 243.22 to 579.58 mg GAE/100 g DW and ABTS antioxidant activity from 487.41 to 904.49 mg TE/100 g DW. Following simulated gastrointestinal digestion, ABTS activity reached 1110.71 mg TE/100 g DW at 10% APC. Increasing APC concentrations reduced protein digestibility from 74.90% to 55.83%, overrun, hardness, and apparent viscosity and progressively modified sorbet color. Sensory evaluation showed the highest acceptance for formulations containing 2.5–7.5% APC, whereas 10% APC reduced sensory quality. Overall, moderate APC incorporation provided an effective balance between nutritional and functional enhancement and sensory acceptability.

## 1. Introduction

Current trends regarding the growing consumer preferences for food products made from natural ingredients, reduced use of synthetic additives and gentler processing conditions have driven the search for new product niches within the food industry. The European frozen food market, which is expected to grow substantially in the coming years, represents such an opportunity [1]. Freezing is widely recognized as an effective method for preserving the sensory and nutritional quality of fruits and vegetables, while frozen fruits and vegetables can serve as valuable raw materials for the production of frozen desserts such as sorbet, whose final quality depends on the formulation and processing conditions [2,3].

Sorbets are non-dairy frozen products characterized by low fat content and a relatively simple formulation, based on fruit, water and sweeteners, typically with total soluble solids of 25–35% to ensure adequate texture and stability. Their technological simplicity, combined with high fruit content and desired sensory properties, makes them suitable carriers for functional ingredients [4]. A wide range of fruits have been explored in sorbet formulations, including apples, which are particularly attractive due to their global availability and nutritional value [5]. Apples offer a balanced composition of water, carbohydrates and dietary fiber (~3 g/100 g), with a predominance of soluble fiber in the form of pectin, a highly esterified and structurally complex polysaccharide. Low in calories, apples provide vitamins, minerals and antioxidants [6].

Recent research has increasingly focused on the development of functional fruit-based frozen desserts through the incorporation of value-added ingredients such as dietary fibers, plant proteins, and bioactive compounds [7]. The addition of soluble fibers, particularly inulin and pectin, has been shown to improve textural properties, water retention, and prebiotic potential while maintaining desirable sensory attributes [8,9]. Moreover, enrichment with plant-derived proteins has gained attention as a strategy to enhance the nutritional profile of frozen desserts, especially in the context of plant-based product development [10]. For example, Malgor et al. added amaranth proteins as a functional ingredient to the manufacture of lemon and amaranth sorbet and following sensory analysis, the product was characterized as having creamy and healthy attributes [11]. The use of aquafaba in frozen desserts made from fruit purees (strawberries, nectarines and bananas) has been shown to be an alternative to animal proteins [12].

In this context, alfalfa protein concentrate (APC) represents a promising, yet underexplored, ingredient for sorbet fortification, offering a combination of high-quality plant protein and biologically active compounds (BAC), with potential benefits for both nutritional enhancement and functional performance [13].

APC derived from fresh green leaves is reported to contain approximately 45–60% protein, including essential amino acids, alongside unsaturated fatty acids, pigments, polyphenolic compounds, vitamins (such as K, B-complex, C, D, E, and folate), and minerals (including calcium, magnesium, iron, copper, and potassium) [14,15]. Moreover, the digestibility of alfalfa proteins is relatively high compared to other plant-based proteins, ranging between 75% and 85% [16]. APC has been utilized as a dietary supplement in countries such as Mexico, Canada, and the United States since 1992, and in 2009 it received a favorable assessment from the European Food Safety Authority, which recommended a daily intake of up to 10 g [17,18].

Fortification of apple sorbet with APC may provide multiple nutritional and functional benefits, as well as important technological implications. From a nutritional perspective, APC can increase the protein content of sorbet, which is naturally low in protein, thereby creating a more balanced product [19]. Additionally, APC contributes essential amino acids, vitamins, and minerals. The elevated levels of BAC, including flavonoids, saponins, and antioxidants, may further support cardiovascular health and immune function [20,21].

From a functional and technological standpoint, APC can improve texture by promoting a creamier structure and reducing ice crystal formation. Its emulsifying and foaming properties may also enhance consistency and mouthfeel [22,23].

Based on these considerations, the present study aimed to develop an apple sorbet enriched with APC as a novel functional food with enhanced nutritional value. The physicochemical, phytochemical and functional properties of APC were first characterized, followed by an evaluation of its effects on the physicochemical characteristics of the sorbet, color, sensory quality, biological value, protein digestibility, rheological behavior and textural properties. In addition, the study aimed to identify the optimal level of APC incorporation that provides the highest nutritional and functional benefits, while maintaining high consumer acceptability.

## 2. Materials and Methods

### 2.1. Chemicals

The chemicals used in this study included 6-hydroxy-2,5,7,8-tetramethylchromane-2-carboxylic acid (Trolox, purity ≥ 97%), 2,2-diphenyl-1-picrylhydrazyl hydrate (DPPH, purity ≥ 95%), and 2,2′-azino-bis(3-ethylbenzothiazoline-6-sulfonic acid) diammonium salt (ABTS, purity ≥ 98%), all purchased from Alpha Aesar (Haverhill, MA, USA). Ninhydrin (≥98%), aluminum chloride hexahydrate (≥98%), quercetin (≥95%) and gallic acid (GA, ≥97%), used as the reference standard, were supplied by Sigma-Aldrich (St. Louis, MO, USA). The Folin–Ciocalteu reagent (2.1 N) was obtained from Chem-Lab NV (Zedelgem, Belgium). Ethanol, *n*-hexane, anhydrous citric acid, concentrated hydrochloric acid, concentrated nitric acid, perchloric acid, and trichloroacetic acid were of analytical or chromatographic grade and were used without further purification. Absorbance measurements were performed with a UV-1900 spectrophotometer (Shimadzu, Tokyo, Japan).

### 2.2. Extraction of Alfalfa Proteins Concentrate (APC) from Medicago sativa *L.*

For this study, a local alfalfa variety, “Dimitra,” cultivated in Talmaza village, Ștefan Vodă district, Republic of Moldova (46°38′09″ N, 29°39′53″ E), was selected as raw material. The aerial parts, located approximately 15 cm from the top of the plant, were collected from different parts of the alfalfa field in May 2025 and stored frozen at −18 ± 1 °C for 21 days before processing. The extraction procedure, with some modifications, was performed according to the method described previously [13].

Without prior thawing, the plant material was weighed and transferred to a laboratory blender (Electrolux E6TB1-6ST, Stockholm, Sweden). Slightly alkaline water (pH 9.0 ± 0.01) was added at a sample-to-solvent ratio of 1:3 (*w*/*v*), with the solvent temperature maintained at 4 ± 1 °C. The alkaline medium was adjusted to pH 9.0 ± 0.01 using a freshly prepared 0.02 M Ca(OH)_2_ solution. Homogenization was carried out at low speed for 1 min per 100 ± 1 g of sample. During the ultrasound-assisted extraction (UAE) process, following homogenization, the samples were subjected to sonication in an ultrasonic bath (ISOLAB Laborgeräte GmbH, Eschau, Bavaria, Germany) operating at a frequency of 37 kHz for 15 min at a temperature of 15 ± 1 °C. The mixtures were then filtered through cotton cloth, and the retained solid fraction was manually pressed to maximize liquid recovery. The filtrates were centrifuged at 10,000 rpm for 10 min at 10 ± 1 °C, after which the supernatants were carefully collected.

Protein precipitation was induced by adjusting the pH of the supernatants to 4.4 ± 0.1 using 1 M citric acid. The samples were subsequently stored at 4 ± 1 °C for 16 h to allow protein precipitation. The resulting suspensions were centrifuged in a refrigerated centrifuge (MPW-380R, MPW Med. Instruments, Warsaw, Poland) using six filled 50 mL Nalgene tubes. Centrifugation was performed at 15,000 rpm for 10 min at 10 ± 1 °C. Following centrifugation, the recovered APC was transferred into sealed containers and stored at −20 ± 1 °C until further analysis. All samples were prepared in triplicate. The extraction yield was 14.62 ± 1.3%, calculated on the dry-weight (DW) basis of the initial alfalfa material. The dry matter content of the resulting APC was 20.03 ± 0.5%.

### 2.3. Analyzing of APC

#### 2.3.1. Physicochemical Analysis

Dry matter was determined by the oven-drying method according to AOAC method 925.10. Protein content was analyzed using the Kjeldahl method (AOAC), with a nitrogen-to-protein conversion factor of 6.25, employing a UDK129 unit (VELP Scientifica, Usmate, Italy). Fat content was determined by Soxhlet extraction (AOAC method 948.22) using a SER148 extraction unit (VELP Scientifica, Monza, Italy), while ash content was measured by dry incineration at 550 ± 1 °C following AOAC method 942.05 (2006). Water activity (a_w_) was measured with a LabSwift-aw analyzer (Novasina AG, Lachen, Switzerland). The pH and titratable acidity, expressed as % citric acid, were determined using a TitroLine 5000 pH meter (SI Analytics, Weilheim, Germany).

#### 2.3.2. Amino Acid Composition

The amino acid composition was determined by cation-exchange chromatography following acid hydrolysis of the sample according to standard analytical procedures, which separate amino acids based on their ionic properties. Quantification of the amino acids was then carried out by AOAC Official Method 982, ensuring accurate and standardized results [24]. Prior to chromatographic analysis, the alfalfa protein concentrate was subjected to acid hydrolysis. Briefly, the sample was hydrolysed with 6 N HCl at 110 °C for 23 h. After hydrolysis, the sample was cooled and filtered, and the hydrolysate was evaporated under reduced pressure at a temperature below 50 °C. The resulting residue was reconstituted in buffer at pH 2.2 prior to chromatographic analysis. The hydrolysed amino acids were separated by cation-exchange chromatography using a column packed with OSTION IG ANB ion-exchange resin (particle size 5–10 µm; column bed height 22 cm). Photometer with wavelength of 570 nm, and flow rate 12 mL per hour. Mobile phase composition: citric acid, lithium citrate, lithium chloride, 2N LiOH, HCl conc., thiodiglycol, ethyl alcohol. Buffer solutions (pH 2.9, pH 2.95, pH 3.2, pH 3.8, pH 5.0). Standard used SIGMA AA-S-18. The total proteinogenic amino acid content was calculated as the sum of the quantified amino acids and expressed as g/100 g DW.

#### 2.3.3. Color Attributes

Sample color was measured using a CR-400 Chroma Meter (Konica Minolta, Osaka, Japan) according to the CIELab color system. The color coordinates included lightness (L*), the red–green axis (a*), and the yellow–blue axis (b*). More negative values of the a*/b* ratio indicate a greater intensity of green coloration [13].

Chroma index (C*) indicates the degree of the color and is proportional to the intensity of the color and was calculated according to the formula [25]:

(1) $$C^{*}=\sqrt{{a^{*}}^{2}+{b^{*}}^{2}}$$

Hue angle (h*) is expressed in a 0–360° range, where 0° = bluish-red, 90° = yellow, 180° = green and 270° = blue. The h* was calculated using the following expression [26]:

(2) $$h^{*}=arctan\left(\frac{b^{*}}{a^{*}}\right)$$

#### 2.3.4. Chlorophyll and Carotenoid Content

The concentrations of chlorophyll a (Chl *a*), chlorophyll b (Chl *b*), total chlorophyll (TChl), and carotenoids (CC) were determined spectrophotometrically following the methanolic extraction procedure described by Sumanta et al. [27]. Pigment concentrations were calculated using the equations reported by Lichtenthaler and Wellburn [27].

The APC samples were analyzed without prior drying. Methanolic extracts were prepared by homogenizing 0.5 g of the wet sample with 10 mL of 99% methanol using a blender (Zepter MixSy; VG-022-K/VO-022-K, Baar, Switzerland) at 10,000 rpm for 1 min at room temperature, while protecting the samples from direct light. The homogenates were then centrifuged at 10,000 rpm for 15 min at 4 ± 1 °C. After centrifugation, the supernatant was carefully collected, and approximately 4.5 mL of 99% methanol was added, corresponding to a nominal total extraction volume of 14.5 mL. The test tubes containing the extracts were wrapped in aluminum foil and stored at 4 ± 1 °C for spectrophotometric determinations. A single extraction cycle was performed. Complete pigment recovery was not independently verified by sequential extraction of the residual material.

Absorbance was measured at 663, 645, and 470 nm for chlorophyll a, chlorophyll b, and carotenoids, respectively, using the spectrophotometer described in Section 2.1. Three independent replicates were analyzed, and each extract was measured in duplicate. Pigment concentrations were expressed on a dry-weight basis (mg/100 g DW), with the dry-weight conversion performed based on the measured dry matter content of the corresponding APC samples.

#### 2.3.5. Total Polyphenols and Flavonoids

For the evaluation of total polyphenol content (TPC), total flavonoid content (TFC), and antioxidant activity (AA), hydroalcoholic extracts were prepared from both APC and sorbet samples. The extraction was performed as follows: the weighed sample was placed in conical flasks, then 70% aqueous ethanol was added in a sample: solvent ratio of 1:50 (*w*/*v*). The flasks were vortexed for 30 min at 30 ± 1 °C, followed by UAE (37 kHz) for 10 min at 25 ± 1 °C. The obtained extracts were subsequently centrifuged at 10,000 rpm for 10 min, and the supernatants were collected and brought to a final volume of 50 mL for further spectrophotometric analysis.

TPC and TFC in APC and sorbet ethanolic extracts were determined spectrophotometrically according to established protocols [28], with minor modifications [29]. TPC were quantified using the Folin–Ciocalteu reagent [30], based on a calibration curve constructed with gallic acid (0–500 mg/L, R^2^ = 0.9978), and the results were expressed as milligrams of gallic acid equivalents per gram of dry weight (mg GAE/g DW).

TFC was assessed using AlCl_3_·6H_2_O, with quantification based on a quercetin standard curve (0–160 mg/L, R^2^ = 0.9974). The results were expressed as milligrams of quercetin equivalents per gram of dry weight (mg QE/g DW).

#### 2.3.6. Radical-Scavenging Activity

AA of the hydroethanolic extracts from APC and sorbet samples was determined following the method described by Paulpriya et al. [31]. Antioxidant activity was expressed as milligrams of Trolox equivalents per gram of dry weight (mg TE/g DW). For the DPPH assay, antioxidant activity was calculated using a Trolox calibration curve prepared over the concentration range of 0–500 µmol/L (R^2^ = 0.9994). All determinations were performed in triplicate. The DPPH assay was conducted using a DPPH• solution prepared in methanol, with an initial concentration of approximately 0.08 mmol/L, adjusted to achieve an initial absorbance of approximately 0.70 at 515 nm. For each determination, 0.40 mL of the hydroethanolic extract or Trolox standard solution was mixed with 2.80 mL of the DPPH• solution. The reaction mixture was incubated for 30 min at room temperature, after which the absorbance was measured at 515 nm. Methanol was used as the blank.

The obtained concentration values were converted to mg TE/g DW according to the following equation, taking into account the total extraction volume (0.05 L), the mass corresponding to 1 μmol of Trolox (0.25029 mg/μmol), and the dry weight of the sample:

(3) $$\frac{mgTE}{g}DW.=\frac{C\left(\frac{\mu mol}{L}\right)*V_{extract}*0.25029}{m_{DW\;sample.}},$$

where

mg TE/g DW—milligrams of Trolox equivalents per gram of dry weight;
*C*—the Trolox-equivalent concentration (μmol/L) obtained from the calibration curve;
*V_extract_*—total volume of the analyzed extract (L);
*m*—the dry weight of the sample.

The ABTS•^+^ radical cation decolorization assay was performed according to the procedure reported by Arnao et al. [32], with antioxidant capacity likewise expressed as mg TE/g DW. The ABTS•^+^ radical cation was generated by mixing 5 mL of 7 mM ABTS solution with 5 mL of 2.45 mM potassium persulfate solution. The mixture was incubated in the dark at room temperature for 12–16 h. Before use, the resulting ABTS•^+^ solution was diluted with ethanol to obtain an absorbance of 0.70 ± 0.02 at 734 nm. For each determination, 0.10 mL of hydroethanolic extract or Trolox standard solution was mixed with 2.40 mL of diluted ABTS•^+^ solution. Absorbance was measured at 734 nm exactly 6 min after mixing, using ethanol as the blank. The Trolox-equivalent concentration, obtained from the calibration curve (0–500 µmol/L, R^2^ = 0.9970), was subsequently converted to mg TE/g DW according to Equation (3).

#### 2.3.7. Foaming Capacity and Foam Stability

Foam capacity (FoC) and foam stability (FoS) of the APC samples were determined according to the method of Cano-Medina et al., with slight modifications. APC dispersions were prepared at a concentration of 20 g/L, and the pH was adjusted to 4.6 using 0.01 M HCl. The samples were homogenized in graduated plastic tubes for 1 min at 3000 rpm using a Vortex V-1 plus mixer (Biosan SIA, Riga, Latvia).

FoC was expressed as a percentage and calculated based on the increase in volume after whipping relative to the initial volume. FoS was evaluated following a similar procedure; however, after foam formation, the samples were allowed to stand for 30 min at room temperature, after which the remaining foam volume was recorded. FoS was expressed as a percentage, calculated as the ratio of the residual foam volume to the total foam volume [33].

#### 2.3.8. Emulsifying Activity and Emulsion Stability

The emulsifying activity (EA) and emulsion stability (ES) of APC samples were evaluated following the method of Wang and Kinsella [34]. Briefly, 0.7 g of protein was dispersed in 10 mL of distilled water and vortexed until fully solubilized. Subsequently, 10 mL of sunflower oil was added, and the mixture was homogenized using a Zepter mixSy (VG-022-K/VO-022-K, Baar, Switzerland) at 16,000 rpm for 1 min to obtain an oil-in-water emulsion.

The emulsion was transferred into 25 mL conical tubes and centrifuged at 3200 rpm for 5 min. Emulsifying activity was expressed as a percentage, calculated as the ratio of the height of the emulsified layer to the total height of the sample in the tube.

For the assessment of emulsion stability, the prepared emulsion was subjected to thermal treatment in a water bath at 80 ± 1 °C for 30 min, followed by cooling to 15 ± 1 °C and subsequent centrifugation. ES was expressed as a percentage, defined as the ratio between the height of the emulsified layer after thermal treatment and the total height of the tube contents.

#### 2.3.9. Evaluation of Textural Properties

The textural properties of the APC sample were determined using a TA.HD Plus texture analyzer (Stable Micro Systems, Surrey, UK). The paste samples—cylindrical in shape with dimensions of 25 × 10 mm—were placed in a suitable container and analyzed at a temperature of 21 ± 1 °C. All measurements were performed in triplicate using a double-compression test with a P/5S stainless steel cylindrical probe. The instrument was equipped with a 50 kg load cell and operated at a pre-test speed of 1.0 mm/s, a test speed of 5.0 mm/s, and a post-test speed of 5.0 mm/s. The probe compressed the sample to a penetration depth of 8 mm, with a trigger force of 10.0 g. The interval between the two compression cycles was 5.0 s. The calculated texture profile parameters included springiness, cohesiveness, and gumminess [13].

### 2.4. Preparation and Analysing of Sorbet with APC

#### 2.4.1. Preparation of Sorbet with APC

Five sorbet samples were obtained, the control sample (CS), without the addition of APC and 4 samples with the addition of APC in the following concentrations: 2.5%, 5.0%, 7.5% and 10.0%.

The apples, the Golden variety, were subjected to reception, washing and removal of impurities, with the aim of eliminating physical and microbial contaminants. Subsequently, the apples were baked at a temperature of 180 ± 1 °C, for 30 ± 1 min, then the inedible parts were removed, and the apple pulp was chopped, resulting in a puree with a moisture of 72 ± 2%. In parallel, the preparation of the sugar and citric acid solution is carried out, dissolved in the amount of water provided for in the manufacturing recipe. The apple puree is mixed with the sugar-citric acid solution and with CPA, at a temperature of 4 ± 1 °C, in the established concentrations, until the components are homogenized. The resulting mix was pasteurized at 84 ± 1 °C for 1–2 min. After pasteurization, the mix was rapidly cooled to 10 ± 1 °C, then matured for 22 ± 2 h at 4 ± 1 °C. Mechanical aeration followed, achieved by mixing for 4 ± 1 min in an ice cream maker (Caso IceCreamer 03298, Arnsberg, Germany), to incorporate air into the mix. The final product was packaged in 50 mL glass jars, then subjected to rapid freezing at –25 ± 1 °C for 15 min. The sorbet was stored at a temperature of –19 ± 1 °C. The manufacturing recipe and sample coding are presented in Table 1.

#### 2.4.2. Physicochemical Analysis

The total solids content was determined by ISO 6731:2010 [35]. Protein content was determined by Kjeldahl methods using a UDK129 automatic Kjeldahl analyzer (VELP Scientifica, Usmate, Italy). The instrument automatically performs the distillation and titration steps and provides the protein content directly as a percentage. A nitrogen-to-protein conversion factor of 6.25 was preset in the instrument for the calculation of protein content. The pH and acidity were measured with a Titrator SI Analytics TitroLine^®^ 5000 (Xylem Analytics, Letchworth, UK), at 20 °C.

#### 2.4.3. Sensory Analysis

The sensory analysis of the sorbet samples was carried out in accordance with ISO 6658:2017 [36]. The sorbet samples were analysed according to the following criteria: consistency, taste and smell appearance, color, by a group of 11 tasters, in tasting booths. The tasters assessed each criterion in points from 0 to 5: 5—exceptional, ideal qualities; 4—appropriate quality; 3—with slight defects; 2—with obvious defects; 1—with strongly pronounced defects; 0—modified, with major changes. Each evaluation criterion was associated with an importance coefficient (*fi*), established according to its relevance in the overall assessment. An equal importance coefficient (*fi* = 0.25) was assigned to each attribute (taste and smell, appearance, consistency, and color).

To apply the method, the average score (*Pm*) for each criterion, the average fractional values (*Pmp*), and the overall average score (*Pmt*) were determined. Each criterion was assigned its own importance coefficient (*fi*), corresponding to its level of relevance. The fractional *Pmp* values were calculated using the following relationship:

(4) Pmp=Pm·fi·ft

where:

*Pm*—arithmetic mean of the scores;
*fi*—importance factor;
*ft*—transformation coefficient.

Thus the 5-point scale used is transformed into a 20-point scale to determine the quality of the product, and *ft* = 4. The overall average score (*Pmt*) was obtained by summing the *Pmp* values corresponding to all evaluation criteria. Depending on this value, the final result of the analysis was established, and the product was assigned a score according to Table 2 [37].

#### 2.4.4. Color Parameters

The color parameters of the sorbet samples, L*, a*, b*, -a*/b* ratio and h* were determined according to the method described in Section 2.3.3.

The total color differences (ΔE*) were calculated between the sorbet samples with ACP and the control sample without ACP addition, using the following formula [38].

(5) $${\Delta E}^{*}=\sqrt{{\left(L_{sample}^{*}-L_{control}^{*}\right)}^{2}+{\left(a_{sample}^{*}-a_{control}^{*}\right)}^{2}+{\left(b_{sample}^{*}-b_{control}^{*}\right)}^{2}}$$

#### 2.4.5. Texture Parameters

The textural properties of the sorbet samples were determined using a TA.HD Plus texture analyzer (Stable Micro Systems, Surrey, UK). A 30 g portion of sorbet was placed in a suitable container and analyzed at a temperature of 1 ± 0.5 °C. All measurements were performed in triplicate using a double-compression test with a P/5S stainless steel cylindrical probe. The instrument was equipped with a 50 kg load cell and operated at a pre-test speed of 1.0 mm/s, a test speed of 5.0 mm/s, and a post-test speed of 5.0 mm/s. The probe compressed the sample to a penetration depth of 8 mm, with a trigger force of 10.0 g. The interval between the two compression cycles was 5.0 s. The calculated texture profile parameters included springiness, cohesiveness, and chewiness.

#### 2.4.6. Overrun of Sorbet

The sorbet overrun was determined using the equal-volume mass method. A calibrated 100 mL container was used to determine the mass of equal volumes of the unfrozen sorbet mix and the frozen sorbet samples. The frozen sorbet samples were analyzed at −18 °C. The mass of the corresponding 100 mL volume of the mix was determined before freezing, and the same volume of the frozen sorbet was subsequently weighed. Overrun was calculated according to the following equation [39]:

(6) $$Overrun\left(\%\right)=\frac{mix\;\left(g\right)-sorbet\;\left(g\right)}{sorbet\;\left(g\right)}\times 100\%$$

#### 2.4.7. Protein Digestibility

Protein digestibility was expressed as a percentage, being calculated as the ratio between the amount of trichloroacetic acid (TCA)-soluble peptides, determined in the supernatant resulting from in vitro digestion, and the total protein content of the analyzed samples, according to Equation (5).

(7) $$Protein\;digestibility\;\left(\%\right)=\frac{B}{A}\times 100\%$$

where:

*A*—total protein content, mg;
*B*—TCA-soluble peptide content, mg [40].

In vitro digestion of sorbet samples was performed according to the INFOGEST 2.0 protocol [41].

A digestion control sample consisting of the apple sorbet matrix without added APC was subjected to the same INFOGEST 2.0 gastrointestinal digestion, sample preparation, and analytical procedures as the APC-containing samples.

The in vitro digestion of samples included a gastric phase involving incubation under continuous agitation for 2 h at 37 °C and pH 3.0, followed by a 2 h intestinal phase at 37 °C and pH 7.0. For subsequent analyses, the supernatant free of insoluble fractions, obtained via centrifugation (17,500× *g* for 10 min), was used. The content of TCA-soluble peptides was analyzed and quantified as mg of bovine serum albumin equivalents per g of sample, according the method of Lowry et al. [42].

#### 2.4.8. Total Polyphenols and Antioxidant Activity in Digests

At the end of the digestion process, the digests were cooled to 5 ± 1 °C and centrifuged at 17,500× *g* for 10 min, at 4 °C to remove insoluble material. The resulting supernatants were collected, and 3 mL of 70% ethanol was added to 2 mL of each supernatant. The addition of ethanol was used to precipitate proteins and other macromolecular components and to facilitate the recovery of phenolic compounds in the resulting ethanolic fraction. The mixtures were then centrifuged at 10,000× *g*, for 5 min at 4 °C, and the resulting supernatants (GID) were collected. A digestion control sample (apple sorbet matrix without added APC) was subjected to the same digestion, extraction, centrifugation, and analytical procedures. The supernatants were stored at −19 °C for 24 h until analysis.

The digests were analyzed for TPC and AA using the Folin–Ciocalteu, DPPH, and ABTS assays, as described in Section 2.3.5 and Section 2.3.6. No further dilution of the samples was necessary. The determinations were performed in triplicate. The results were expressed on the basis of the dry weight of the original sample subjected to in vitro digestion.

#### 2.4.9. Rheological Analysis of Sorbet

The rheological behavior of the sorbet samples was evaluated using a controlled-stress rotational rheometer, HAAKE MARS (Thermo Scientific, Karlsruhe, Germany). Measurements were performed using a parallel-plate geometry consisting of an 80 mm diameter lower plate and a 40 mm diameter upper rotor. To ensure precise data acquisition and minimize slip effects, a gap of 1 mm was maintained between the plates for all experiments. The temperature was strictly controlled at 20 °C throughout the analysis using a Peltier temperature control system.

After loading the sample between the plates and adjusting the measurement gap, the sample was allowed to equilibrate for 600 s before the rheological measurement was initiated. The same equilibration procedure was applied to all samples. The rheological measurement was subsequently performed for 10 min.

**Flow behavior and apparent viscosity measurements**: The apparent viscosity (η) was determined under a constant shear rate to characterize the flow behavior and consistency of the sorbet matrices. This approach follows the methodology described by Goff et al. for evaluating the structural resistance of frozen desserts [43].

**Dynamic Rheological Analysis (Frequency Sweep):** Oscillatory frequency sweep tests were performed to characterize the viscoelastic behavior of the sorbet samples. Measurements were conducted over a frequency range of 0.1–100 Hz under identical experimental conditions for all formulations. The storage modulus (G′) and loss modulus (G″) were recorded as functions of frequency to evaluate the elastic and viscous components, respectively, of the viscoelastic response of the sorbet matrices. The frequency dependence of G′ and G″ was compared among the control and APC-enriched sorbets. The predominance of G′ over G″ throughout the investigated frequency range indicated a predominantly elastic (solid-like) response under the applied measurement conditions [44].

#### 2.4.10. Fourier Transform Infrared Spectroscopy (FTIR) Analysis of Sorbet Samples

The chemical composition and structural fingerprints of the sorbet samples were analyzed using a Fourier Transform Infrared Spectrometer (Nicolet iS50, Thermo Scientific, Waltham, MA, USA), equipped with a diamond attenuated total reflectance accessory. The spectra were recorded in the mid-infrared region, ranging from 4000 to 400 cm^−1^, at a resolution of 4 cm^−1^. To ensure a high signal-to-noise ratio, each spectrum was obtained by averaging 32 scans. Before each measurement, a background spectrum was collected using the clean, dry diamond crystal under ambient conditions. Prior to ATR-FTIR analysis, the sorbet samples were removed from low-temperature storage and allowed to thaw at room temperature for 15 min. The thawed samples were subsequently subjected to spectral analysis under identical experimental conditions. A sufficient amount of sample was placed directly onto the ATR crystal to completely cover its active measurement surface. The sample was spread evenly across the crystal surface, and the same procedure was followed for all measurements. The crystal was thoroughly cleaned and dried between successive measurements. Each sorbet formulation was measured three times using the same sample under identical experimental conditions. Consequently, these triplicate measurements represented technical replicates (repeated instrumental measurements of the same sample) rather than independently prepared samples. Baseline correction was applied to all acquired spectra using the instrument’s software to minimize baseline variations and facilitate sample comparison. The same correction procedure was consistently applied to all spectra, the spectra were corrected using the instrument’s software OMNIC™. The analysis focused on the characteristic bands of the sorbet matrix, specifically the regions associated with O-H stretching (water and sugars, 3600–3000 cm^−1^) and the fingerprint region (1200–900 cm^−1^) related to the vibrations of soluble solids and carbohydrates, following the methodology proposed by Kamińska-Dwórznicka et al. and Spinei et al. for emulsion systems [45,46].

### 2.5. Statistical Analysis

All measurements were conducted in triplicate, and the results are presented as the mean ± standard deviation (SD) of three independent determinations. Preliminary data processing and calculations were performed using Microsoft Office Excel 2007 (Microsoft, Redmond, WA, USA). Statistical analyses were carried out using Statgraphics Centurion XVI, version 16.1.17 (Statgraphics Technologies, Inc., The Plains, VA, USA). The effect of APC supplementation at five inclusion levels (0, 2.5, 5.0, 7.5, and 10.0%, *w*/*w*) on the measured response variables of the sorbet samples was evaluated using one-way analysis of variance (ANOVA). When significant effects were observed, differences among means were determined using Tukey’s honestly significant difference (HSD) post hoc test. Statistical significance was established at *p* ≤ 0.05. Principal component analysis (PCA) was performed using the Scikit-learn library (version 1.3.2) in Python (version 3.10) to evaluate the relationships among the physicochemical properties, texture profile parameters, biologically active compounds, CIELab color parameters, and sensory attributes of the APC-enriched sorbet samples.

## 3. Results and Discussion

### 3.1. Characterization of APC

The physicochemical properties, color attributes, phytochemical content, antioxidant activity, qualitative and quantitative amino acid profile, and textural and functional properties of APC extracted from *Medicago sativa* L. from the local alfalfa cultivar Dimitra were determined and analyzed (Table 3, Table 4, Table 5 and Table 6).

Ultrasound-assisted aqueous extraction was used to enhance the release of soluble proteins from homogenized alfalfa biomass. The APC yield was 14.62% based on DW, which is lower than the value reported in the literature (approximately 25% DW) for protein concentrate obtained from freshly pressed alfalfa leaf juice [47,48]. The authors of the present study previously reported that several factors may contribute to the relatively low APC yield, including the use of an aqueous extraction solvent and the nature of the raw material, specifically the aerial parts of alfalfa (leaves and stems) collected from the upper 15 cm of the plant [13]. It has been reported that alfalfa stems contain approximately two times less protein than leaves [49,50]. The protein content of the APC obtained in this study was 86.45% on a dry weight basis. This value is comparable in magnitude to that reported for alfalfa protein isolates obtained from leaves via intensified extraction technologies. For example, a yield of 14.5 g/100 g DW and a protein content of 91.1 g/100 g were reported for an alfalfa protein isolate produced through an ultrasound-assisted alkaline isoelectric precipitation and ultrafiltration (UUAAIP) process under optimized conditions [51].

The ash and fat content of APC is relatively low, but these fall within the values indicated in the bibliography [15,47].

The titratable acidity, calculated in relation to citric acid (1.58%), is higher compared to that determined for frozen alfalfa (0.56%), and the pH of APC (4.16) is also lower compared to that of frozen alfalfa (5.84) [13]. The increase in acidity is due to processing and ultrasound treatment, which affects the structure of the plant material and destroys the cell walls, facilitating the penetration of the solvent and the release of organic acids and polyphenols from the plant matrix [52].

The CIELab color parameters indicated that APC exhibited a characteristic yellow–green color typical of leaf protein concentrates (Table 3). Its intermediate lightness (L* = 52.16), strongly negative a* value (−23.14), and positive b* value (38.72) reflected the predominance of chlorophylls and carotenoids retained after extraction [53]. The high chroma (C* = 45.11) indicated intense color saturation, while the hue angle (120.9°) confirmed the characteristic yellow–green appearance of the concentrate.

Spectrophotometric analysis of hydroalcoholic extracts of APC revealed the presence of considerable amounts of BAC, including chlorophylls and other chloroplastic constituents, carotenoids, polyphenols, flavonoids, and related phytochemicals (Table 2). These compounds may interact with alfalfa proteins, thereby influencing, to varying extents, their digestibility as well as their functional and antioxidant properties [54,55].

The low pigment content observed in the APC can be attributed, at least in part, to the extraction conditions employed, specifically, the use of a hydrophilic solvent. The stability and retention of chlorophylls and carotenoids can be affected by several factors, including pH, as well as exposure to light, oxidizing agents and ultrasound treatment [56,57].

In particular, considerable amounts of polyphenols and flavonoids were quantified in APC, which can be attributed to the cavitation effects induced by UAE. The AA of APC, evaluated using the DPPH and ABTS assays, showed high values, namely 519.8 and 1443.7 mg TE/100 g DW, respectively.

These findings indicate that the incorporation of APC into food matrices can enhance their biological and functional value, mainly through the contribution of proteins and associated bioactive constituents. Table 4 presents a summary of the qualitative and quantitative amino acid profile of the APC, determined via cation-exchange chromatography. The Kjeldahl method indicated a protein content of 86.45% in the APC (Table 3), whereas the total content of proteinogenic amino acids, determined following acid hydrolysis, was 56.28 g/100 g DW. The difference between these values stems from two analytical approaches based on distinct principles and methodologies. In the Kjeldahl procedure, protein content was estimated based on total nitrogen using a nitrogen-to-protein conversion factor of 6.25. In contrast, amino acid analysis involves acid hydrolysis followed by chromatographic separation, identification, and quantification; the resulting values can be influenced by hydrolysis efficiency, amino acid recovery rates, and the specific analytical response of each amino acid.

The predominant amino acids identified in APC were consistent with those commonly found in alfalfa, including aspartic acid, glutamic acid, leucine, lysine, and alanine (Table 4). These amino acids are generally present in higher concentrations in dried alfalfa (hay) [58,59]. Tryptophan was not determined under the applied acid-hydrolysis conditions and was therefore not included in the reported amino acid profile. It has been reported that its concentration in alfalfa is generally low, approximately 0.15–0.20 mg/100 g DM [55,59]. Nevertheless, the protein concentrate contains a favorable proportion of essential amino acids, with a total of 28.76 g/100 g DW APC, which contributes to its nutritional and functional value.

The textural properties of the APC samples, as determined in this study, are presented in Table 5.

Texture parameters provide a quantitative characterization of the mechanical and structural properties of the obtained APC samples. A previous study [13] comparatively evaluated APC samples obtained via conventional extraction and ultrasound-assisted extraction, highlighting differences in their functional and structural properties. The results indicated that ultrasound-extracted APC formed a denser and more stable protein network, as reflected by higher values for hardness and gumminess. In this context, Gul et al. demonstrated that ultrasonic treatment improves the homogeneity, rigidity, and elasticity of plant protein networks [60].

Table 6 shows the functional properties of APC sample. Foaming properties are important functional characteristics of proteins used in food formulations, as they contribute to the formation and stabilization of aerated structures, thereby potentially influencing the texture and sensory characteristics of the final product. APC exhibited a foaming capacity of 84.36% and a foam stability of 85.41%, demonstrating a high capacity to generate and maintain foam under the conditions used in this study. Protein performance in foaming is generally correlated with the ability to migrate and adsorb at the air-water interface, to alter conformation or rearrange at the interface, and to form interfacial layers capable of stabilizing the incorporated air bubbles [61,62]. The foaming properties observed in the present study are considered experimental functional characteristics of the APC, while the underlying interfacial mechanisms remain to be verified through complementary structural and physicochemical analyses.

The emulsifying properties of APC (Table 6) indicate its ability to form relatively stable emulsions, with EA and ES values within the ranges reported in the literature for alfalfa proteins, suggesting that APC exhibits surfactant properties comparable to those of other food proteins [34]. In addition, APC was obtained using UAE, which may contribute to its emulsifying properties, as previous studies have reported enhanced emulsification performance of ultrasound-treated animal- and plant-derived proteins compared with their untreated counterparts [63].

### 3.2. Characterization of Sorbet Samples

#### 3.2.1. The Sensory Properties of the Sorbet Samples

**Figure 1:**
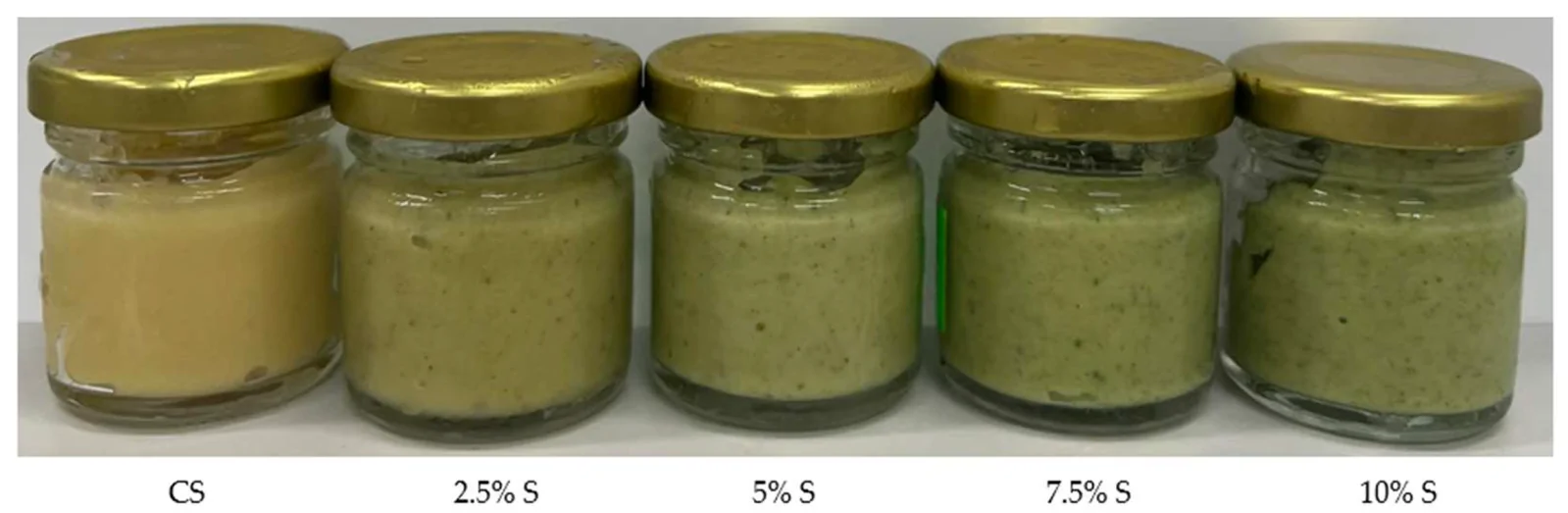
Samples of sorbet: CS—control sample, without APC; 2.5% S—sorbet with 2.5% APC; 5% S—sorbet with 5% APC; 7.5% S—sorbet with 7.5% APC; 10% S—sorbet with 10% APC.

Sensory analysis is essential for evaluating consumer perceptions of food products, as it quantifies their acceptability during the development of new products or the optimization of existing ones [64]. The results of the sensory analysis as well as images of the sorbet samples are presented in Table 7 and Figure 1, respectively.

One-way analysis of variance (ANOVA) revealed a significant effect of APC level on the analyzed sensory parameters (F(4,10) = 45.47, *p* < 0.001) for sorbet samples. Samples containing 2.5–7.5% APC achieved the highest scores and did not differ significantly from each other (*p* > 0.05), indicating excellent consumer perception. These findings demonstrate that moderate APC incorporation does not adversely affect sensory attributes, preserving optimal taste and odor, appearance, consistency, and color. In addition, compared to the CS, which exhibited a slightly lower consistency score (4.55), APC addition improved textural properties, likely due to enhanced water-binding capacity and structural stabilization [49].

In contrast, the formulation with 10% APC showed significantly lower values than the 2.5–7.5% treatments (*p* < 0.05), indicating that excessive APC addition negatively affected sensory quality, resulting in a lower total score (18.67) and overall sensory profile (4.66). These declines can be attributed to the more pronounced vegetal (alfalfa-like) flavor and alterations in texture and color at higher protein levels. Overall, moderate APC incorporation (2.5–7.5%) provided the most favorable results.

#### 3.2.2. Physicochemical Analysis of the Sorbet Samples

The results of the physicochemical indicators, TPC, TFC and AA were presented in Table 8.

One-way ANOVA and Tukey’s HSD test (*p* < 0.05) showed that the incorporation of APC significantly influenced the DM content, protein content, pH, and titratable acidity of the sorbets (Table 8). DM increased progressively from 27.43% in the control sample to 34.63% in the variant with 10% APC, representing a 26.3% increase. This rise aligns with the higher solids contribution from APC and indicates that APC incorporation substantially altered the solid-to-water ratio of the variants. Protein content showed a much more pronounced, concentration-dependent increase, from 0.11% in CS to 8.05% at 10% APC, confirming APC efficacy as a protein-enriching ingredient.

APC addition also resulted in a significant, albeit comparatively modest, increase in pH, rising from 3.27 in CS to 3.79 at 10% APC (*p* = 0.018). However, only the variant with 10% APC exhibited significant differences compared to the control. This response can be attributed to the buffering capacity of the proteins and minerals introduced via APC [65]. In contrast, titratable acidity increased from 6.47 in CS to 7.44 at 10% APC (*p* < 0.001), with significant differences becoming particularly evident at higher supplementation levels. The simultaneous increase in pH and titratable acidity indicates that APC modified the acid-base buffering characteristics of the sorbet matrix, rather than simply increasing or decreasing its acidity.

No significant differences in a_w_ were detected among the variants (*p* > 0.05). Thus, despite the increase in solids and protein content, the incorporation of APC did not result in a measurable change in the thermodynamic availability of water under the conditions of this experiment. This finding is relevant because it indicates that the compositional changes induced by APC addition were not accompanied by a corresponding shift in a_w_ values.

Protein digestibility decreased progressively from 74.90% in the CS to 55.83% in the variant with 10% APC (*p* < 0.001), with significant differences observed across all supplementation levels. The progressive decline in protein digestibility as the level of APC inclusion increased could be partly attributed to the higher protein load introduced into the digestive system. As the proportion of APC rose, so did the amount of protein available as a substrate for digestive enzymes, while the conditions for enzyme activity remained constant. Consequently, the enzyme-to-substrate ratio may have decreased, thereby limiting the extent of protein hydrolysis and resulting in a lower percentage of digested protein. Furthermore, differences in the structural and physicochemical characteristics of the plant-based proteins introduced via APC could have influenced their susceptibility to enzymatic hydrolysis [19,66]. These factors may cumulatively contribute to the lower protein digestibility observed at higher APC levels, although the relative contribution of each mechanism was not directly investigated in this study. However, since these interactions were not directly investigated in the present study, they should be considered as possible causes rather than proven mechanisms.

An opposite effect was observed regarding the overrun, which decreased from 62.74 ± 1.96% in the CS to 45.12 ± 1.88% in the variant with 10% APC (*p* < 0.001). Significant reductions were observed at APC levels of 7.5% and 10%, whereas no significant differences were recorded between the control sample and the variants with 2.5% and 5% APC. The decrease in overrun may reflect changes in the composition and flow behavior of the sorbet matrix, driven by the higher dry matter and protein content of the APC, factors that can influence air incorporation during freezing and agitation. Similar reductions in overrun have been reported for protein-enriched frozen desserts [67]. It is worth noting that the absence of significant differences at lower APC levels suggests that the effect becomes noticeable only after a certain degree of fortification is exceeded.

Incorporation of phytochemical-rich APC significantly increased the TPC and TFC values of the sorbets (Table 8) in a concentration-dependent manner (Tukey’s HSD test, *p* < 0.05). Relative to the CS, TPC and TFC increased by approximately 1.9-fold and 1.5-fold, respectively, in the 2.5% S, reaching approximately 2.4-fold and 2.9-fold higher values, respectively, in the 10% S.

The increase in phytochemical content was accompanied by enhanced AA. ABTS radical scavenging activity increased approximately 1.6-fold and 2-fold at APC concentrations of 7.5% and 10%, respectively, compared to the control sample (*p* < 0.001). DPPH activity also increased significantly following APC incorporation (*p* < 0.001), with values rising from 210.23 to 287.30 mg TE/100 g DW across the range of supplementation. The differing magnitudes of the responses obtained via the ABTS and DPPH methods indicate that the antioxidant contribution of APC depends on the analytical method employed. Consequently, the results support the notion of increased overall radical scavenging capacity in the fortified sorbets, though they should not be interpreted as a direct measure of the concentration or activity of individual antioxidant compounds.

Overall, APC incorporation resulted in correlated changes in the composition and functional properties of the sorbets: increasing APC levels led to higher dry matter, protein, TPC, TFC, and AA values, while simultaneously reducing protein digestibility and the degree of expansion (overrun). In contrast, water activity remained unchanged, whereas pH and titratable acidity showed more moderate variations. These results indicate that APC fortification simultaneously alters multiple aspects of sorbet functionality and that the magnitude of these changes depends on the level of incorporation.

The products resulting from the simulated gastric and intestinal digestion (GID) of the sorbet samples were analyzed to determine TPC and AA using the Folin–Ciocalteu and DPPH/ABTS methods, respectively (Table 8). Since the digestion process generates a complex matrix containing phenolic compounds, peptides, amino acids, and other substances with reducing potential or the ability to neutralize free radicals, the responses obtained via these methods cannot be considered specific solely to phenolic compounds [68,69]. A sorbet without APC was subjected to the same simulated gastrointestinal digestion procedure and served as a control sample.

Despite the changes induced by digestion, statistical analysis revealed a highly significant effect of APC supplementation on TPC and AA measured by both the ABTS and DPPH methods (*p* < 0.001). Tukey’s HSD test indicated significant differences between supplementation levels (*p* < 0.05), with higher APC concentrations generally leading to higher TPC values and AA in the GID material.

Spectrophotometric analyses revealed a decrease in TPC (GID), values dropped approximately twofold for the formulation with 10% APC and 4.5-fold for the one with 2.5% APC. Similarly, DPPH (GID) values decreased approximately twofold across all formulations after digestion. The reduction in TPC (GID) may be attributed to the degradation, transformation, or reduced extractability of phenolic compounds under gastrointestinal conditions [70]. These changes may also contribute to the lower DPPH response observed post-digestion. However, the DPPH method is not specific to phenolic compounds, and the measured can also be influenced by other reducing compounds present in the digested material [71].

In contrast, ABTS (GID) values decreased after digestion for the CS samples and those with 2.5% APC, whereas an approximately 1.2-fold increase was observed in formulations containing 5–10% APC. This divergent response between the two methods may reflect differences in the reactivity of compounds generated or released during digestion. Specifically, gastrointestinal hydrolysis can release peptides and amino acids with antioxidant properties, as well as other reducing compounds, which may contribute to the ABTS response [68]. The broader reactivity of the ABTS radical cation toward both hydrophilic and lipophilic compounds may also contribute to the difference in response observed compared to the DPPH method.

#### 3.2.3. CIELab Color Analysis of Sorbet Samples

In fruit-based frozen desserts, visual characteristics are often associated with sensory quality and the presence of natural ingredients. The development of a characteristic yellow-green coloration following the addition of APC may serve as a visual indicator of the incorporation of plant-derived functional components.

The CIELab color parameters demonstrated that APC addition significantly modified the appearance of apple sorbet, Table 9.

The L* parameter was significantly affected by APC incorporation (*p* < 0.05). The CS exhibited the highest lightness value (69.03), indicating the brightest appearance among the formulations. Tukey HSD analysis confirmed significant differences between CS and all APC-enriched samples (*p* < 0.05). The addition of 2.5% APC resulted in a marked decrease in L* (51.53), followed by a further reduction with increasing APC concentration, reaching 34.98 in the 10% S. The 5% S and 7.5% S did not differ significantly (*p* > 0.05), forming the same homogeneous group. The reduction in lightness with increasing APC concentration can be associated with the presence of natural pigments in APC, mainly chlorophylls and carotenoids, which intensify coloration and decrease perceived brightness.

The a* parameter was also significantly influenced by APC addition (*p* < 0.001). The CS showed the highest a* value (−4.65), reflecting a weaker contribution of green tones compared with APC-containing samples. According to Tukey HSD analysis, CS differed significantly from all APC-enriched samples (*p* < 0.05). The 2.5% S exhibited a significantly lower a* value (−11.17) than the CS and differed from the 5% S, 7.5% S, and 10% S samples (*p* < 0.05). No significant differences were observed among the latter three samples (*p* > 0.05), which formed a common homogeneous group. The shift in a* values toward more negative values indicated an increased contribution of green coloration, primarily associated with chlorophyll pigments introduced through APC. These results demonstrate that APC addition promoted the development of green tones, with the most pronounced effect occurring at concentrations above 5%.

The b* parameter showed a significant response to APC incorporation (*p* < 0.001). The CS presented the highest b* value (40.36), indicating the strongest contribution of yellow tones originating from the apple puree matrix. All APC-enriched samples differed significantly from the CS (*p* < 0.05). The 2.5% S showed an intermediate b* value (35.29) and differed significantly from the remaining samples. No significant differences were observed between the 5% S and 7.5% S samples (*p* > 0.05), whereas the 10% S exhibited the lowest b* value (26.53) and differed significantly from all other samples (*p* < 0.05). The decrease in b* values indicate a reduction in yellow intensity as APC concentration increased, suggesting that alfalfa-derived pigments progressively altered the balance between yellow and green chromatic components.

The −a*/b* ratio was significantly affected by APC addition (*p* < 0.001). The CS had the lowest value (0.11), whereas the ratio increased with APC concentration, reaching 0.56 in the 10% S sample. Assuming negative a* values and positive b* values, this increase indicates a stronger green component relative to the yellow component. Tukey’s HSD test showed significant differences among CS, 2.5% S, and 10% S, while 5% S and 7.5% S did not differ significantly (*p* > 0.05).

The h* was significantly influenced by APC incorporation (*p* < 0.001). The CS exhibited the lowest h* value (96.6°), corresponding to a predominantly yellow hue typical of apple-based sorbet. The 2.5% S showed an intermediate h* (107.6°) and differed significantly from both the CS and higher APC samples (*p* < 0.05). The 5% S and 7.5% S samples formed the same homogeneous group (*p* > 0.05), whereas the 10% S formulation exhibited the highest h* value (119.3°), differing significantly from all other samples (*p* < 0.05). The increase in h* values indicate a transition from yellow toward yellow-green coloration, resulting from the increasing influence of alfalfa pigments on the original color profile of the apple sorbet.

The ΔE* increased significantly with APC concentration (*p* < 0.001), demonstrating the progressive modification of sorbet appearance. The 2.5% S showed a significantly higher ΔE* value (19.35) compared with CS, indicating that even a low APC addition produced a perceptible color change. However, ΔE* values for the 5–10% S samples were significantly higher than those of 2.5% S (*p* < 0.05). The 5% S and 7.5% S samples did not differ significantly (*p* > 0.05), while the 10% S sample showed the highest ΔE* value (38.17), significantly differing from all other samples (*p* < 0.05). The increase in ΔE* reflects the cumulative effect of APC on lightness reduction, increased greenness, and hue modification. The observed color differences indicate that APC enrichment produces clearly distinguishable visual changes compared with the CS, which may influence consumer perception of the product [72].

#### 3.2.4. Rheological Behavior of Sorbet

**Figure 2:**
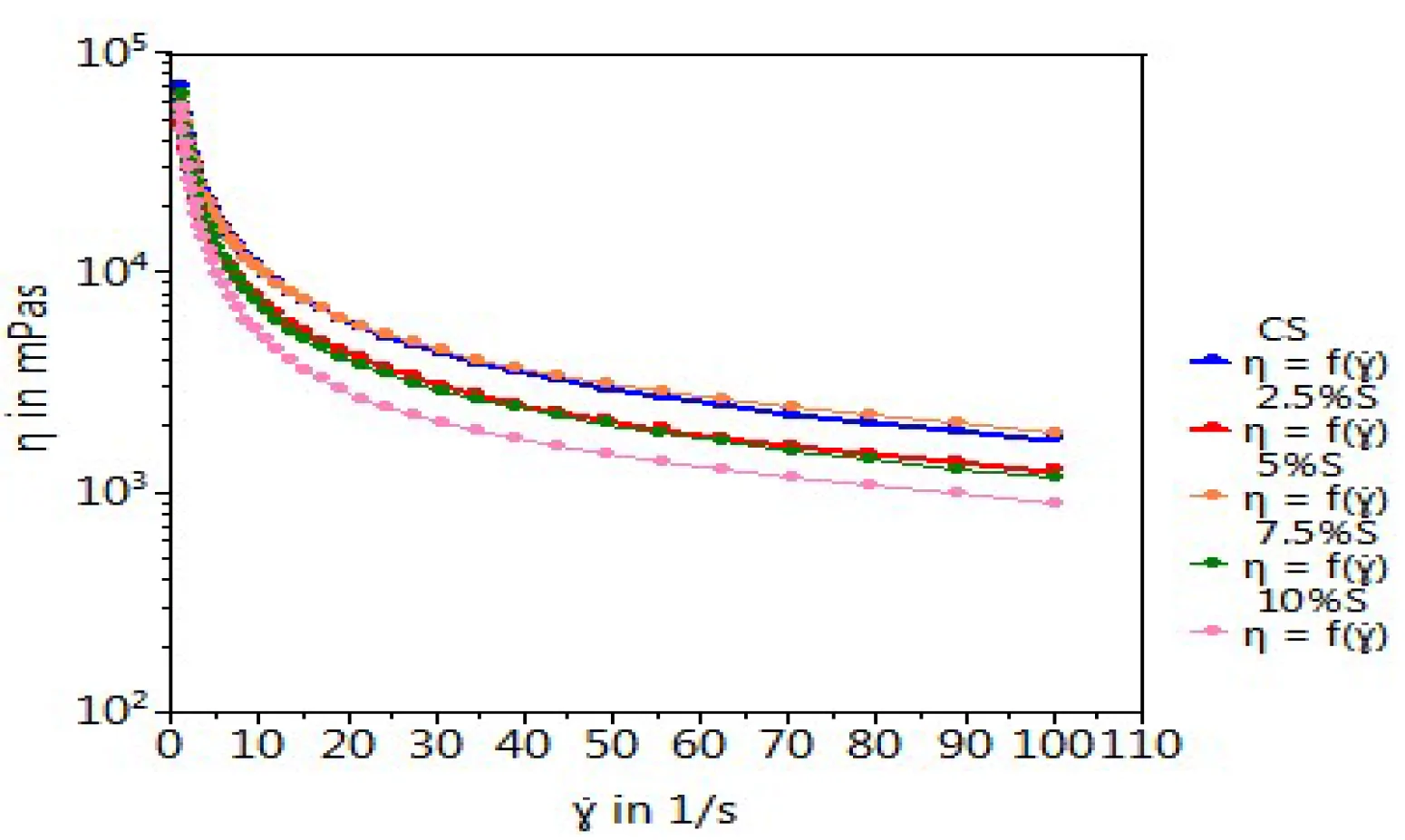
The flow behavior of the sorbet formulations: CS—control sample, without APC; 2.5% S—sorbet with 2.5% APC; 5% S—sorbet with 5% APC; 7.5% S—sorbet with 7.5% APC;10% S—sorbet with 10% APC.

The evolution of apparent viscosity (η) as a function of shear rate (γ) for the sorbet samples is shown in Figure 2. All formulations showed non-Newtonian pseudoplastic behavior (shear-thinning), characterized by a substantial decrease in viscosity with increasing shear rate. This phenomenon is typical for sorbet and ice cream, being attributed to the orientation of biopolymer molecules and the deformability of ice crystals and air bubbles in the flow direction, which reduces the internal resistance of the matrix.

A clear influence of the APC concentration on the rheological profile was observed. The CS presented the highest viscosity values over the entire shear range (0–100 s^−1^), suggesting a denser structural network and stronger intermolecular interactions in the absence of the addition. Increasing the concentration of APC from 2.5% to10% led to a progressive and systematic decrease in viscosity (CS > 2.5% S > 5% S > 7.5% S > 10% S). Although the protein content of the sorbets progressively increased from 0.11% in CS to 8.05% in 10% S, APC incorporation generally decreased the apparent viscosity compared with the control. This apparently inverse relationship indicates that the rheological effect of APC was governed not only by protein concentration but also by the structural and functional characteristics of the protein-rich concentrate and its interactions with the other constituents of the sorbet matrix. APC was characterized by a high protein content (86.45%). The protein concentrate was particularly rich in glutamic acid, leucine, lysine, aspartic acid, and alanine. The presence of amino acids with polar and/or ionizable side chains, particularly glutamic acid, aspartic acid, and lysine, indicates the availability of functional groups capable of participating in hydration, electrostatic interactions, and hydrogen bonding. However, these molecular characteristics did not result in a proportional increase in the apparent viscosity of the sorbet matrix, suggesting that protein–protein and protein–matrix associations were more important in determining the rheological response than a simple thickening effect. Therefore, increasing the APC concentration did not increase the resistance of the system to flow; instead, it may have altered the connectivity and mobility of the continuous phase, resulting in lower apparent viscosity values than those observed for CS. Changes in the acid–base characteristics may also have contributed to modifications in protein–matrix interactions. The pH increased from 3.27 in CS to 3.79 in 10% S, while titratable acidity increased from 6.47 to 7.44%, expressed as citric acid. The simultaneous changes in pH, acidity, and protein concentration suggest that APC modified the chemical environment of the continuous phase. Since protein hydration, intermolecular association, and protein–polysaccharide interactions are pH-dependent, these compositional changes may have contributed to the progressive decrease in apparent viscosity observed with increasing APC concentration.

**Figure 3:**
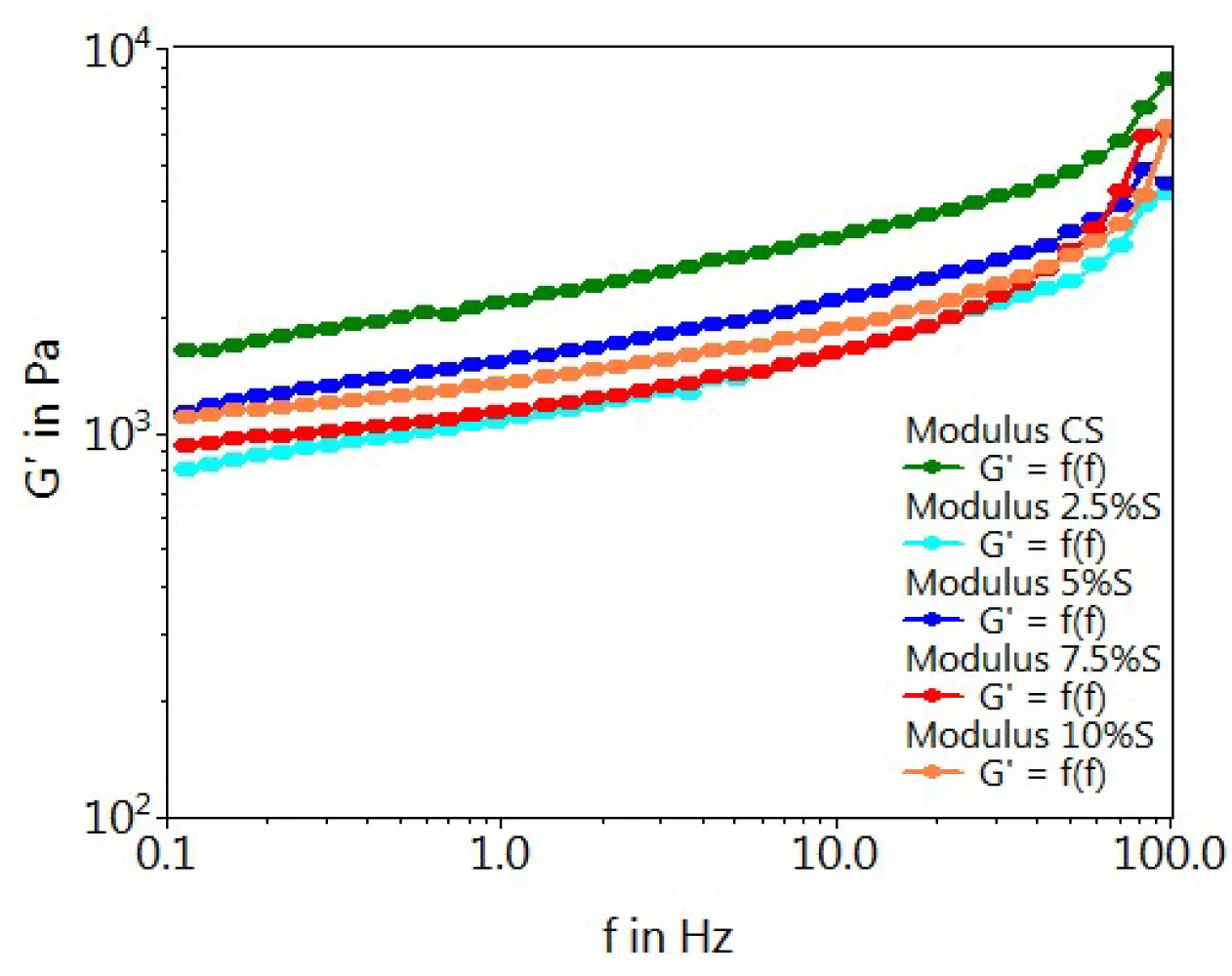
The storage modulus of the sorbet formulations: CS—control sample, without APC; 2.5% S—sorbet with 2.5% APC; 5% S—sorbet with 5% APC; 7.5% S—sorbet with 7.5% APC; 10% S—sorbet with 10% APC.

It highlighted the viscoelastic behavior of the sorbet samples over a frequency range from 0.1 to 100 Hz. The increase in modulus (G′) with frequency suggests that the sorbet behaved like a weak gel, where the structure was stable but dependent on the time scale of deformation (Figure 3).

**Figure 4:**
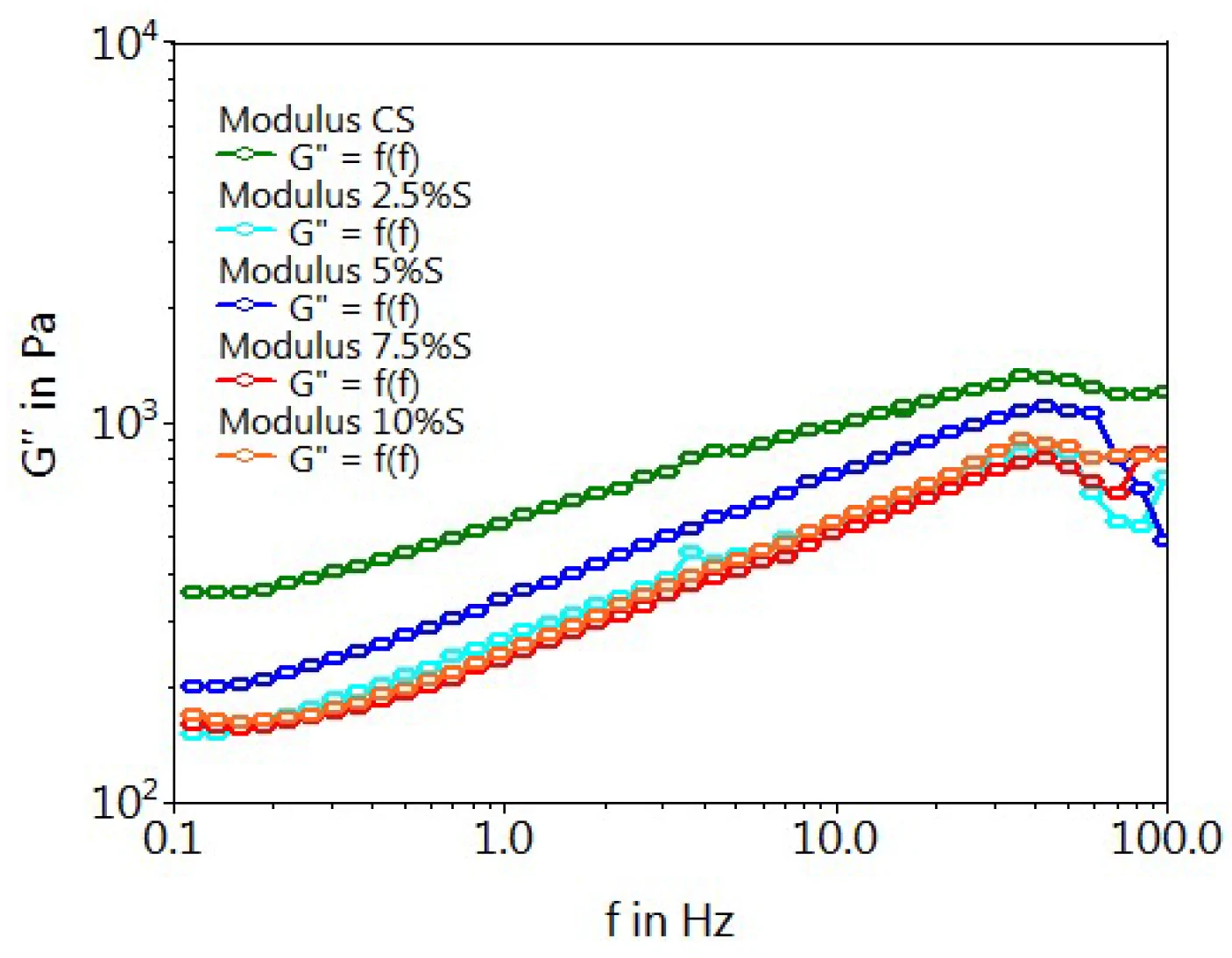
The loss modulus of the sorbet formulations: CS—control sample, without APC; 2.5% S—sorbet with 2.5% APC; 5% S—sorbet with 5% APC; 7.5% S—sorbet with 7.5% APC; 10% S—sorbet with 10% APC.

The storage modulus (G′) revealed differences in the elastic response of the sorbet formulations. CS exhibited the highest G′ values, indicating the greatest resistance to reversible deformation and, consequently, a more strongly interconnected elastic structure. APC incorporation generally resulted in a decrease in G′ compared with CS, indicating that the protein concentrate modified the organization of the structural network responsible for the elastic response. This behavior is particularly relevant considering that the dry matter content progressively increased with increasing APC concentration. The decrease in the elastic modulus cannot be attributed to dilution of the solid components responsible for structure formation. The results indicate that the increase in total dry matter content resulting from APC addition did not necessarily lead to the formation of a stronger elastic network. Therefore, the rheological response appears to depend to a greater extent on the nature, interactions, and structural integration of the added components than on the total dry matter content. The high protein content of APC and the ability of these proteins to interact with water and other matrix constituents may have altered the organization of the continuous phase. Once incorporated into the sorbet matrix, APC proteins and associated components may interfere with the interactions responsible for maintaining the original elastic network, introducing structural discontinuities and modifying water distribution and the interactions between the continuous phase and dispersed structural elements. The lower G′ values observed following APC incorporation indicate that the concentrate induced reorganization rather than reinforcement of the elastic network of the sorbet. Therefore, the rheological behavior cannot be explained solely by the increase in protein or dry matter content but should be interpreted in the context of changes in matrix organization and connectivity induced by APC incorporation. The evolution of the parameter G″ as a function of frequency is represented in Figure 4. The viscous modulus provided information about the ability of the sorbet to flow and form a specific gel structure. Comparing the loss modulus curves with those of the storage modulus (G′) in the previous figure, it is observed that G′ > G″ over the entire frequency range. This confirms that the sorbet with added APC has a predominantly elastic, solid-type behavior, specific to weak gels. Similar to the elastic modulus, the control CS presented the highest values of G″, indicating a higher internal resistance to deformation. The addition of APC led to a decrease in the viscous component. Interestingly, samples 2.5% S and 7.5% S presented the lowest values of G″, suggesting a more aerated structure or a weaker interaction between the component phases.

All samples showed an increase in G″ with frequency, which indicates that, at high deformation rates, energy dissipation increases. This trend is linear up to approximately 50 Hz, where fluctuations are observed. The sudden decrease and the “peak” observed at very high frequencies of 80 Hz could indicate a structural breakage phenomenon or exceeding the stability limit of the plate-plate geometry under high inertia forces. The decrease in the values for the modulus G″ by the addition of APC suggests that the samples become less “viscous” in the dynamic regime, which, corroborated the decrease in G′, indicates the obtaining of a sorbet with a more malleable texture and easier processing compared to the CS, which for a frozen dessert with aeration properties is a beneficial characteristic.

#### 3.2.5. FTIR Characteristics of Sorbet

**Figure 5:**
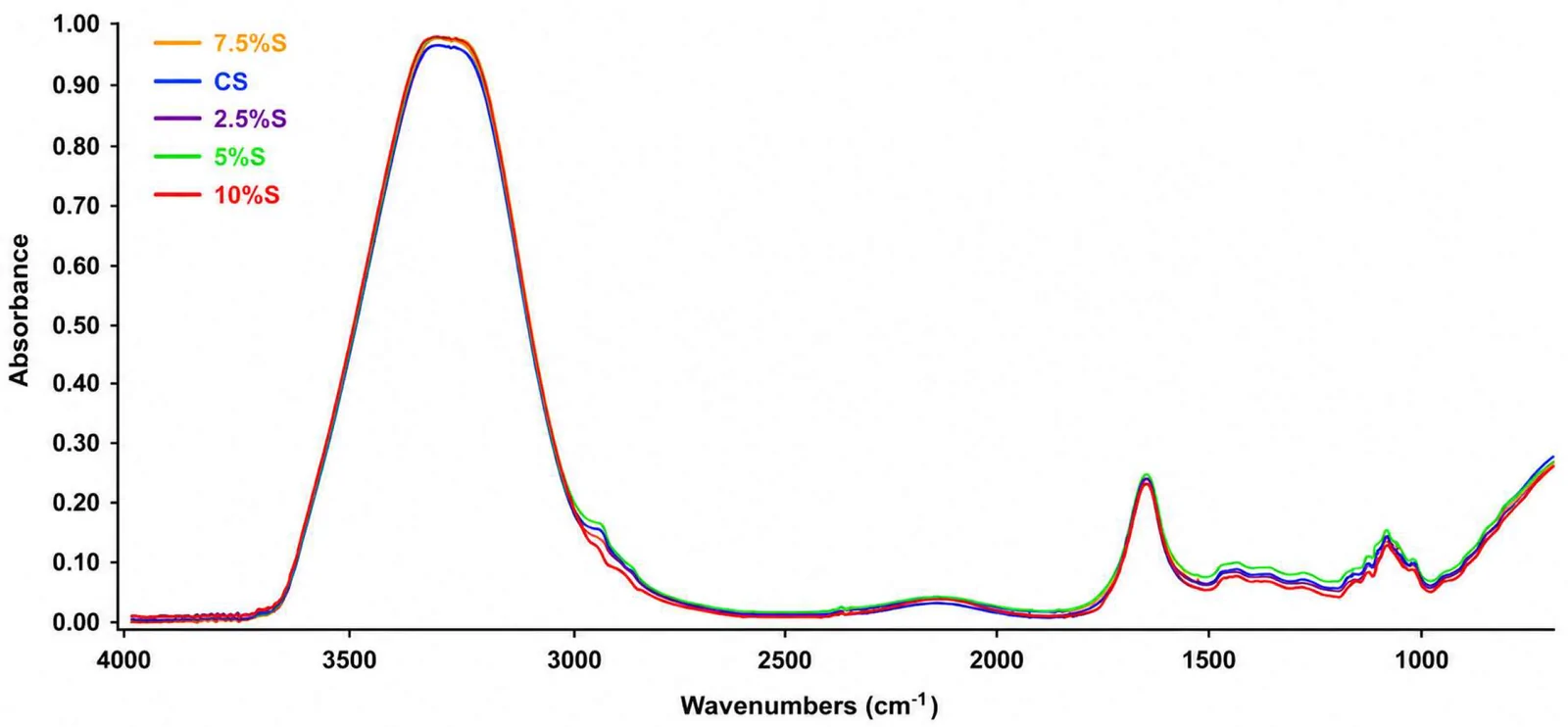
FTIR characterization of sorbet samples: CS—control sample, without APC; 2.5% S—sorbet with 2.5% APC; 5% S—sorbet with 5% APC; 7.5% S—sorbet with 7.5% APC; 10% S—sorbet with 10% APC.

The interpretation of the spectra reveals the persistence of the fundamental signals of the addition of APC, indicating a remarkable stability of the organic matrix, Figure 5.

The ATR-FTIR spectra of the CS and APC-enriched sorbets (2.5–10%) showed highly similar spectral profiles, dominated by the characteristic absorption bands of the aqueous carbohydrate-rich matrix. The broad and intense band centered at approximately 3340 cm^−1^ was mainly assigned to O–H stretching vibrations associated with water and hydroxyl-containing constituents. The bands observed in the 2850–2950 cm^−1^ region were attributed to aliphatic C–H stretching vibrations. A distinct band was observed around 1640 cm^−1^, mainly associated with H–O–H bending vibrations. In the fingerprint region (approximately 1200–900 cm^−1^), the spectral features were mainly related to C–O and C–C stretching vibrations of carbohydrate constituents, with the prominent signal around 1060 cm^−1^ being consistent with C–O/C–O–C vibrations characteristic of carbohydrate structures.

The incorporation of APC at concentrations ranging from 2.5 to 10% did not result in the appearance of additional absorption bands or conspicuous shifts or disappearance of the major characteristic bands. Therefore, within the sensitivity of ATR-FTIR analysis, APC incorporation did not produce major detectable changes in the principal functional-group profile of the sorbet matrix. Although slight differences in band intensity were observed among formulations, these variations were not interpreted as evidence of specific molecular interactions, since quantitative interpretation would require appropriate spectral normalization and statistical validation. FTIR was used as a complementary qualitative technique to evaluate the overall spectral fingerprint of the sorbet formulations following APC incorporation rather than as a stand-alone method for demonstrating specific molecular interactions or discriminating among formulations.

#### 3.2.6. Texture Profile Analysis of Sorbet Samples

The results regarding the texture parameters of the analyzed sorbet samples are presented in Table 10.

Hardness decreased significantly from 796.56 ± 14.11 g in the CS to 232.14 ± 18.26 g in the sorbet containing 10% APC, representing a reduction of approximately 71%, (F(4,10) = 1126.0; *p* < 0.001). Tukey’s HSD post-hoc test confirmed significant differences among all sorbet samples (*p* < 0.05), demonstrating a progressive decrease in hardness with increasing APC concentration. This effect can be attributed to the high hydration and water-holding capacity of alfalfa proteins, which limits the formation of a compact structural network and large ice crystals, resulting in a less rigid structure and a softer texture [4]. The consistent reduction in hardness demonstrates that APC influences the textural properties of sorbet in a dose-dependent manner.

Adhesiveness decreased significantly by approximately 71%, from 884.85 ± 10.35 g·s in the CS to 255.19 ± 12.87 g·s in the sample 10% S, (F(4,10) = 772.2; *p* < 0.001). Tukey’s HSD post-hoc test confirmed significant differences among all samples (*p* < 0.05). This behavior can be attributed to the ability of alfalfa proteins to modify the structure of the frozen matrix and reduce product stickiness through a more uniform distribution of water and weaker interactions between the unfrozen phase and the contact surface. These results demonstrate that increasing the APC concentration produces sorbets with progressively lower adhesiveness and less sticky texture.

The results for cohesiveness of sorbet samples were significantly influenced by the addition of APC (F(4,10) = 119.0; *p* < 0.001). Tukey’s HSD post-hoc test indicated significant differences among most experimental samples (*p* < 0.05), except between the sorbets containing 5% and 7.5% APC, which did not differ significantly (*p* > 0.05). This behavior suggests that, at moderate concentrations, alfalfa proteins promote the formation of a more integrated internal network, thereby increasing the matrix’s resistance to deformation. At higher APC levels, however, this effect appears to stabilize, resulting in no further significant improvement in cohesiveness.

The gumminess of the sorbet samples was significantly influenced by the addition of APC (F(4,10) = 45.03; *p* < 0.001). The post-hoc Tukey HSD test showed that CS presented a significantly higher gumminess than all APC-enriched samples (*p* < 0.05), while the 10% S presented the lowest gumminess, significantly different from all other samples. Overall, the reduction in gumminess may be attributed to the combined decrease in hardness and changes in matrix cohesion associated with the high water-holding capacity of alfalfa proteins, potentially promoting the formation of a less compact frozen structure. Consequently, APC supplementation results in sorbets with a less dense texture that deform more readily under applied force.

The chewiness parameter of the sorbet samples was decreasing, with APC intake having a significant effect (F(4,10) = 99.56; *p* < 0.001). The post-hoc Tukey HSD test showed that CS presented a significantly higher chewiness than all APC-enriched sorbets (*p* < 0.05), while the sorbet containing 10% APC recorded the lowest value, significantly different from all other samples. In addition, the samples with 2.5% and 7.5% APC did not differ significantly from each other (*p* > 0.05), as they belonged to the same statistical group. This trend can be explained by the concomitant reduction in hardness and gummy taste with increasing APC concentration, resulting from changes in the sorbet matrix structure and the increased water-binding capacity of alfalfa proteins. Consequently, sorbets enriched with APC require less chewing effort, providing a softer texture and improving ease of consumption.

### 3.3. Relationships Between Physicochemical Characteristics, Texture Profile Analysis, Biologically Active Compounds, CIELab Color and Sensory Parameters in Sorbet with APC

**Figure 6:**
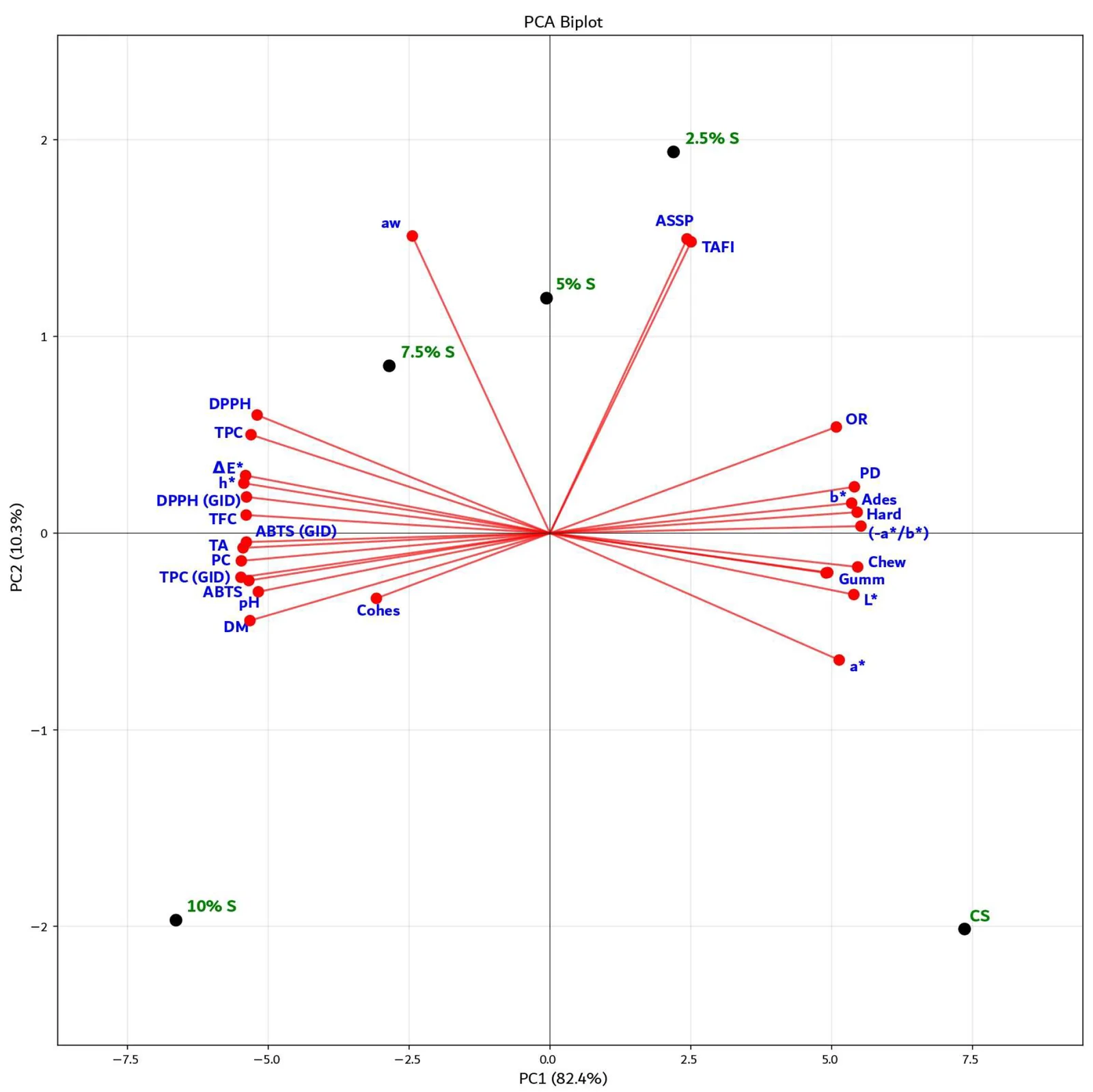
Principal component analysis: CS—control sample, without APC; 2.5% S—sorbet with 2.5% APC; 5% S—sorbet with 5% APC; 7.5% S—sorbet with 7.5% APC; 10% S—sorbet with 10% APC; DM—dry matter; PC—protein content; pH—active acidity; TA—titratable acidity; a_w_—water activity; PD—protein digestibility; OR—overrun; TPC—total polyphenol content; TFC—total flavonoid content; ABTS—2,2-azino-bis-3-ethylbenzothiazoline-6-sulphonic acid; DPPH—2,2-diphenyl-1-picrylhydrazyl; TPC (GID)—total polyphenol content after simulated gastric and intestinal digestion; DPPH (GID)—antioxidant activity determined using the DPPH assay after simulated gastric and intestinal digestion; ABTS (GID)—antioxidant activity determined using the ABTS assay after simulated gastric and intestinal digestion; Hard—hardness; Adhes—adhesivity; Cohes—cohesiveness; Gumm—gumminess; Chew—chewiness; L*—lightness; a*—red–green parameter; b*—yellow–blue parameter; −a*/b*—greenness index; h*—hue angle; ΔE*—total color difference; TAFI—total average score, taking into account factors of importance; ASSP—average score of the sensory profile.

Principal component analysis (PCA) was applied as an exploratory multivariate approach to visualize general patterns among the physicochemical, antioxidant, textural, and color characteristics of the sorbet formulations, Figure 6. The first two principal components accounted for 92.7% of the total variance, with PC1 explaining 82.4% and PC2 10.3%. Given the limited number of formulations relative to the number of variables included in the analysis, the PCA was interpreted descriptively and was not used to infer robust multivariate grouping or causal relationships.

The exploratory PCA showed that the formulations were distributed mainly along PC1. CS was located at the positive extreme of PC1, whereas 10% S occupied the opposite negative region. The 7.5% S formulation was also positioned on the negative side of PC1, while 2.5% S and 5% S were located in the positive or central region and showed positive PC2 scores. This distribution appears to reflect, at least partly, the increasing APC concentration and the associated variation in the measured quality parameters. The negative side of PC1 was associated mainly with TPC, TFC, ABTS, DPPH, and their GID-related parameters, whereas several textural and color variables were oriented toward the positive side. A similar pattern was observed in the correlation matrix (Figure S1). TPC showed strong positive correlations with TFC (*r* = 0.93), ABTS (*r* = 0.92), DPPH (*r* = 1.00), DPPH (GID) (*r* = 0.95), and ABTS (GID) (*r* = 0.94). Strong positive associations were also observed among the GID-related parameters. These relationships are consistent with the similar orientation of the corresponding vectors in the PCA biplot and suggest coordinated variation among the phenolic, flavonoid, and antioxidant parameters. The position of 10% S, and to a lesser extent 7.5% S, in the direction of the antioxidant and phenolic variables suggests that the higher APC levels were more closely associated with this group of parameters. Water activity (a_w_) showed a somewhat different contribution, with a positive PC2 loading and moderate positive correlations with TPC (*r* = 0.66) and DPPH (*r* = 0.70), contributing to the differentiation of the 7.5% S formulation. In contrast, several textural and color characteristics were oriented toward the positive side of PC1, opposite the antioxidant and phenolic variables. For example, hardness was negatively correlated with TPC (*r* = −0.95), TFC (*r* = −0.99), ABTS (*r* = −0.99), and DPPH (*r* = −0.93). Color parameters also contributed to the observed pattern: L* was negatively associated with TPC (*r* = −0.98), ABTS (*r* = −0.95), and DPPH (*r* = −0.97), whereas h* and ΔE* showed positive associations with antioxidant-related variables. PC2 provided additional descriptive differentiation, particularly through a_w_, TAFI, and ASSP. The similar orientation of the TAFI and ASSP vectors was consistent with their strong positive correlation (*r* = 1.00), and both were positioned in the direction of the 2.5% S formulation. In addition, PD and OR were positioned on the positive side of PC1 and were strongly positively correlated (*r* = 0.98), while showing inverse associations with several antioxidant-related parameters. Overall, the exploratory PCA, considered together with the correlation matrix, suggests that increasing APC concentration was associated with coordinated variation in several antioxidants, physicochemical, textural, and color characteristics of the sorbets. The pronounced separation between CS and 10% S represents contrasting multivariate profiles, while the intermediate APC concentrations showed transitional patterns. Considering the limited number of formulations relative to the number of variables, the PCA findings should be regarded as exploratory and used primarily to describe general patterns and associations among the investigated parameters.

## 4. Conclusions

The results demonstrate the potential of APC obtained by the UAE as a multifunctional ingredient for protein-enriched frozen desserts. APC contained 86.45% protein (DW), 28.76 g/100 g DW essential amino acids, and substantial amounts of polyphenols (2297.4 mg GAE/100 g DW) and flavonoids (983.6 mg QE/100 g DW), together with high antioxidant activity and favorable foaming and emulsifying properties.

APC incorporation (2.5–10%) significantly improved the nutritional and bioactive profile of apple sorbet. Protein content increased from 0.11% in the control to 8.05% at 10% APC, while TPC increased from 243.22 to 579.58 mg GAE/100 g DW and ABTS antioxidant activity from 487.41 to 904.49 mg TE/100 g DW. Following simulated gastrointestinal digestion, APC supplementation significantly influenced antioxidant responses, with ABTS activity reaching 1110.71 mg TE/100 g DW at 10% APC.

Increasing APC concentration also modified the technological properties of the sorbet, reducing overrun, apparent viscosity, hardness, adhesiveness, gumminess, and chewiness, while producing progressively darker and greener coloration. Hardness decreased from 796.56 to 232.14 g, whereas protein digestibility decreased from 74.90% to 55.83%, indicating a trade-off associated with higher APC levels.

Sensory evaluation showed the highest acceptance for formulations containing 2.5–7.5% APC, whereas 10% APC significantly reduced sensory quality. Overall, the 2.5–7.5% APC range provided the most favorable balance between nutritional and functional enrichment, technological characteristics, and sensory acceptability, supporting APC as a promising plant-derived ingredient for value-added frozen desserts.

## Figures and Tables

**Table 1:** Manufacturing recipe and sample coding of Sorbet.

Ingredients, %	Sorbet Samples				
	CS	2.5% S	5% S	7.5% S	10% S
Apple puree	86.5	84.0	81.5	79.0	76.5
Sugar	6	6	6	6	6
Citric acid salt	0.5	0.5	0.5	0.5	0.5
Water	7	7	7	7	7
APC	-	2.5	5.0	7.5	10.0

CS—control sample, without APC; 2.5% S—sorbet with 2.5% APC; 5% S—sorbet with 5% APC; 7.5% S—sorbet with 7.5% APC; 10% S—sorbet with 10% APC.

**Table 2:** The grade given to the product according to the overall average score.

Overall Average Score (*Pmt*)	Note
18.1–20.0	Very good
15.1–18.0	Good
11.1–15.0	Satisfactory
7.1–11.0	Unsatisfactory
0–7.0	Inadequate

**Table 3:** Physicochemical indices, BAC, AA and CIELab color parameters of the APC (the results are expressed as means ± standard deviations of three experiments).

Indices	Quantity
Physicochemical Indices	
Dry mater content, %	20.03 ± 0.5
Protein content, % DW	86.45 ± 1.62
Ash content, % DW	0.51 ± 0.01
Fat content, % DW	1.26 ± 0.05
Titratable acidity, % expressed in citric acid	1.58 ± 0.01
pH	4.16 ± 0.01
a_w_, c.u.	0.771 ± 0.002
CIELab color parameters	
L*	52.16 ± 0.11
a*	−23.14 ± 0.16
b*	38.72 ± 0.28
−a*/b*	0.60 ± 0.07
C*	45.11 ± 0.23
h*, °	120.9 ± 0.6
Biologically active compounds	
Chl *a*, mg/100 g DW	4.94 ± 0.09
Chl *b*, mg/100 g DW	2.06 ± 0.02
TChl, mg/100 g DW	6.98 ± 0.05
CC, mg/100 g DW	0.73 ± 0.01
TPC, mg GAE/100 g DW	2297.4 ± 2.7
TFC, mg QE/100 g DW	983.6 ± 3.8
ABTS AA, mg TE/100 g DW	1443.7 ± 4.1
DPPH AA, mg TE/100 g DW	519.8 ± 1.6

a_w_—water activity; L*—lightness; a*—red–green parameter; b*—yellow–blue parameter; −a*/b*—greenness index; C*— chromaticity; h*—hue angle; Chl *a*—chlorophylls *a*; Chl *b*—chlorophylls *b*; TChl—total chlorophylls; CC—carotenoid content; TPC—total polyphenol content; TFC—total flavonoid content; ABTS—2,2-azino-bis-3-ethylbenzothiazoline-6-sulphonic acid; AA—antioxidant activity; DPPH—2,2-diphenyl-1-picrylhydrazyl; GAE—gallic acid equivalents; QE—quercetin equivalents; TE—trolox equivalents; DW—dry weight.

**Table 4:** Amino acid compositions of the APC used for experiments (the results are expressed as means ± standard deviations of three experiments).

Amino Acids, g/100 g DW	Value
Aspartic acid	4.79 ± 0.04
Threonine *	3.68 ± 0.02
Serine	2.74 ± 0.04
Glutamic	7.10 ± 0.02
Proline	1.53 ± 0.02
Glycine	3.11 ± 0.03
Alanine	4.23 ± 0.07
Valine *	3.65 ± 0.05
Cysteine	0.55 ± 0.01
Methionine *	0.81 ± 0.01
Isoleucine *	2.11 ± 0.02
Leucine *	5.83 ± 0.06
Tyrosine	3.16 ± 0.03
Phenylalanine *	3.82 ± 0.05
Lysine *	4.83 ± 0.04
Histidine *	1.85 ± 0.01
Arginine	1.85 ± 0.03
Ammonia	0.36 ± 0.01
∑TAAs	56.28 ± 0.27
∑INM	56.03 ± 0.41
∑NEAAs	27.48 ± 0.18
∑EAAs	28.76 ±0.39
∑IAAs	26.95 ± 0.31
∑GAAs	22.24 ± 0.28
∑AAsK	19.86 ± 0.18
∑AAsP	56.28 ± 0.51
∑AA sulfur containing	1.38 ± 0.08

* essential amino acids; ∑TAAs—the total amino acids; ∑INM—indicators of nitrogen metabolism; ∑NEAAs—non-essential amino acids; ∑EAAs—essential amino acids; ∑IAAs—immunoactive amino acids; ∑GAAs—glycogen amino acids; ∑AAsK—amino acids ketogenic; ∑AAsP—amino acids proteinogenic.

**Table 5:** Texture parameters of APC sample.

Parameters	APC Sample
Hardness, g	38.72 ± 0.43
Adhesivity, g·s	45.59 ± 0.76
Springiness	1.003 ± 0.002
Cohesiveness	0.488 ± 0.006
Gumminess, g	17.16 ± 0.21

**Table 6:** Functional properties of APC sample.

Indices	APC Sample
FoC, %	84.36 ± 0.94
FoS, %	85.41 ± 0.62
EA, %	53.77 ± 0.41
ES, %	55.31 ± 0.28

FoC—foam capacity; FoS—foam stability; EA—emulsifying activity; ES—emulsifying stability.

**Table 7:** Sensory characteristics of the sorbet with APC (results are presented as mean ± standard deviation).

Sensory Characteristics	Sorbet Samples				
	CS	2.5% S	5% S	7.5% S	10% S
Total average score, taking into account factors of importance	19.55 ± 0.13 ^b^	20.00 ± 0.0 ^a^	20.00 ± 0.0 ^a^	20.00 ± 0.0 ^a^	18.67 ± 0.31 ^c^
The average score of the sensory profile	4.88 ± 0.07 ^b^	5.0 ± 0.0 ^a^	5.0 ± 0.0 ^a^	5.0 ± 0.0 ^a^	4.66 ± 0.04 ^c^
Taste and odor	5.0 ± 0.0 ^a^	5.0 ± 0.0 ^a^	5.0 ± 0.0 ^a^	5.0 ± 0.0 ^a^	4.50 ± 0.03 ^b^
Appearance	5.0 ± 0.0 ^a^	5.0 ± 0.0 ^a^	5.0 ± 0.0 ^a^	5.0 ± 0.0 ^a^	5.0 ± 0.0 ^a^
Consistency	4.55 ± 0.04 ^b^	5.0 ± 0.0 ^a^	5.0 ± 0.0 ^a^	5.0 ± 0.0 ^a^	4.50 ± 0.02 ^c^
Color	5.0 ± 0.0 ^a^	5.0 ± 0.0 ^a^	5.0 ± 0.0 ^a^	5.0 ± 0.0 ^a^	4.65 ± 0.04 ^b^

CS—control sample, without APC; 2.5% S—sorbet with 2.5% APC; 5% S—sorbet with 5% APC; 7.5% S—sorbet with 7.5% APC; 10% S—sorbet with 10% APC; different letters (^a–c^) indicate significant differences (*p* < 0.05).

**Table 8:** Physicochemical indicators, a_w_, BAC and AA of the sorbet with APC (results are presented as mean ± standard deviation).

Indicators	Sorbet Samples				
	CS	2.5% S	5% S	7.5% S	10% S
DM, %	27.43 ± 0.43 ^d^	28.30 ± 0.65 ^c,d^	29.97 ± 1.00 ^c^	32.23 ± 0.82 ^b^	34.63 ± 0.65 ^a^
Protein content, %	0.11 ± 0.01 ^e^	2.14 ± 0.12 ^d^	4.01 ± 0.13 ^c^	6.13 ± 0.18 ^b^	8.05 ± 0.29 ^a^
pH	3.27 ± 0.14 ^b^	3.47 ± 0.10 ^a,b^	3.43 ± 0.13 ^a,b^	3.51 ± 0.22 ^a,b^	3.79 ± 0.14 ^a^
Titratable acidity, % expressed in citric acid	6.47 ± 0.05 ^c^	6.77 ± 0.12 ^b,c^	6.90 ± 0.12 ^b^	7.33 ± 0.21 ^a^	7.44 ± 0.09 ^a^
a_w_, c.u.	0.732 ± 0.017 ^a^	0.741 ± 0.003 ^a^	0.739 ± 0.013 ^a^	0.738 ± 0.017 ^a^	0.737 ± 0.025 ^a^
PD, %	74.90 ± 0.92 ^a^	70.97 ± 0.61 ^b^	66.83 ± 1.04 ^c^	59.90 ± 1.90 ^d^	55.83 ± 2.20 ^e^
Overrun, %	62.74 ± 1.96 ^a^	61.47 ± 1.60 ^a^	59.45 ± 1.95 ^a^	49.82 ± 1.57 ^b^	45.12 ± 1.88 ^b^
TPC, mg GAE/100 g DW	243.22 ± 1.69 ^e^	450.82 ± 0.68 ^d^	473.12 ± 1.97 ^c^	542.44 ± 0.72 ^b^	579.58 ± 2.35 ^a^
TFC, mg QE/100 g DW	111.13 ± 1.65 ^e^	166.77 ± 0.91 ^d^	217.20 ± 2.00 ^c^	284.37 ± 1.29 ^b^	318.20 ± 1.70 ^a^
ABTS AA, mg TE/100 g DW	487.41 ± 0.88 ^e^	596.35 ± 0.82 ^d^	677.24 ± 1.05 ^c^	765.33 ± 0.89 ^b^	904.49 ± 0.72 ^a^
DPPH AA, mg TE/100 g DW	210.23 ± 1.10 ^c^	262.20 ± 0.95 ^b^	264.53 ± 1.30 ^b^	283.23 ± 1.08 ^a^	287.30 ± 1.15 ^a^
TPC (GID), mg GAE/100 g DW	64.37 ± 0.25 ^e^	100.10 ± 1.00 ^d^	172.73 ± 0.55 ^c^	256.37 ± 0.95 ^b^	298.33 ± 1.15 ^a^
ABTS (GID), mg TE/100 g DW	220.51 ± 1.25 ^e^	501.57 ± 1.21 ^d^	853.41 ± 1.29 ^c^	910.33 ± 0.87 ^b^	1110.71 ± 1.19 ^a^
DPPH (GID), mg TE/100 g DW	71.31 ± 1.15 ^e^	123.37 ± 0.97 ^d^	175.44 ± 0.67 ^c^	183.64 ± 0.99 ^b^	212.69 ± 0.80 ^a^

CS—control sample, without APC; 2.5% S—sorbet with 2.5% APC; 5% S—sorbet with 5% APC; 7.5% S—sorbet with 7.5% APC; 10% S—sorbet with 10% APC; DM—dry matter; pH—active acidity; a_w_—water activity; PD—protein digestibility; TPC—total polyphenol content; TFC—total flavonoid content; ABTS—2,2-azino-bis-3-ethylbenzothiazoline-6-sulphonic acid; AA—antioxidant activity; DPPH—2,2-diphenyl-1-picrylhydrazyl; TPC (GID)—total polyphenol content after simulated gastric and intestinal digestion; DPPH (GID)—antioxidant activity determined using the DPPH assay after simulated gastric and intestinal digestion; ABTS (GID)—antioxidant activity determined using the ABTS assay after simulated gastric and intestinal digestion; GAE—gallic acid equivalents; QE—quercetin equivalents; TE—trolox equivalents; DW—dry weight; different letters (^a–e^) indicate significant differences (*p* < 0.05).

**Table 9:** CIELab color parameters of sorbet samples.

Color Parameters	Sorbet Samples				
	CS	2.5% S	5% S	7.5% S	10% S
L*	69.03 ± 0.04 ^a^	51.53 ± 0.45 ^b^	45.50 ± 1.38 ^c^	44.00 ± 0.44 ^c^	34.98 ± 0.85 ^d^
a*	−4.65 ± 0.42 ^a^	−11.17 ± 0.61 ^b^	−13.40 ± 0.81 ^c^	−14.28 ± 0.69 ^c^	−14.91 ± 0.90 ^c^
b*	40.36 ± 0.51 ^a^	35.29 ± 0.66 ^b^	33.45 ± 0.81 ^c^	33.26 ± 0.67 ^c^	26.53 ± 0.62 ^d^
−a*/b*	0.11 ± 0.01 ^a^	0.32 ± 0.01 ^b^	0.40 ± 0.01 ^c^	0.43 ± 0.01 ^c^	0.56 ± 0.05 ^d^
h*, °	96.6 ± 0.5 ^d^	107.6 ± 0.6 ^c^	111.8 ± 0.8 ^b^	113.2 ± 0.6 ^b^	119.3 ± 2.0 ^a^
ΔE*	-	19.35 ± 0.40 ^c^	26.05 ± 1.25 ^b^	27.74 ± 0.37 ^b^	38.17 ± 1.17 ^a^

CS—control sample, without APC; 2.5% S—sorbet with 2.5% APC; 5% S—sorbet with 5% APC; 7.5% S—sorbet with 7.5% APC; 10% S—sorbet with 10% APC; L*—lightness; a*—red–green parameter; b*—yellow–blue parameter; −a*/b*—greenness index; h*—hue angle; ΔE*—total color difference; different letters (^a–d^) indicate significant differences (*p* ≤ 0.05).

**Table 10:** Texture parameters of sorbet samples.

Texture Parameters	Sorbet Samples				
	CS	2.5% S	5% S	7.5% S	10% S
Hardness, g	796.56 ± 14.11 ^a^	611.68 ± 12.51 ^b^	486.42 ± 11.08 ^c^	384.15 ± 7.84 ^d^	232.14 ± 18.26 ^e^
Adhesivity, g.s	884.85 ± 10.35 ^a^	733.79 ± 11.57 ^b^	534.80 ± 12.90 ^c^	393.69 ± 11.14 ^d^	255.19 ± 12.87 ^e^
Cohesiveness	0.141 ± 0.002 ^c^	0.111 ± 0.003 ^d^	0.175 ± 0.003 ^a^	0.174 ± 0.004 ^a^	0.165 ± 0.004 ^b^
Gumminess, g	110.61 ± 2.37 ^a^	68.02 ± 3.07 ^c^	89.69 ± 4.23 ^b^	66.34 ± 12.33 ^c^	47.17 ± 3.57 ^d^
Chewiness, g	110.93 ± 2.23 ^a^	68.58 ± 3.59 ^c^	89.08 ± 4.60 ^b^	67.51 ± 5.96 ^c^	47.32 ± 3.68 ^d^

CS—control sample, without APC; 2.5% S—sorbet with 2.5% APC; 5% S—sorbet with 5% APC; 7.5% S—sorbet with 7.5% APC; 10% S—sorbet with 10% APC; different letters (^a–e^) indicate significant differences (*p* < 0.05).

## Data Availability

The original contributions presented in this study are included in the article and Supplementary Material. Further inquiries can be directed to the corresponding author.

## Supplementary Materials

The following supporting information can be downloaded at https://www.mdpi.com/article/10.3390/foods15193504/s1, Figure S1: Feature correlation heatmap.
