# Supplementary material for: Assessment of the Physicochemical, Textural, Sensory, and Antioxidant Properties of Sorbet Enriched with Alfalfa Protein Concentrate
Source: Foods. 2026 Sep 30;15(19):3504. doi: 10.3390/foods15193504 (PMC13664633; doi:10.3390/foods15193504)
Supplement: Supplementary file 1 [file foods-15-03504-s001.zip › foods-4571371-supplementary.pdf]

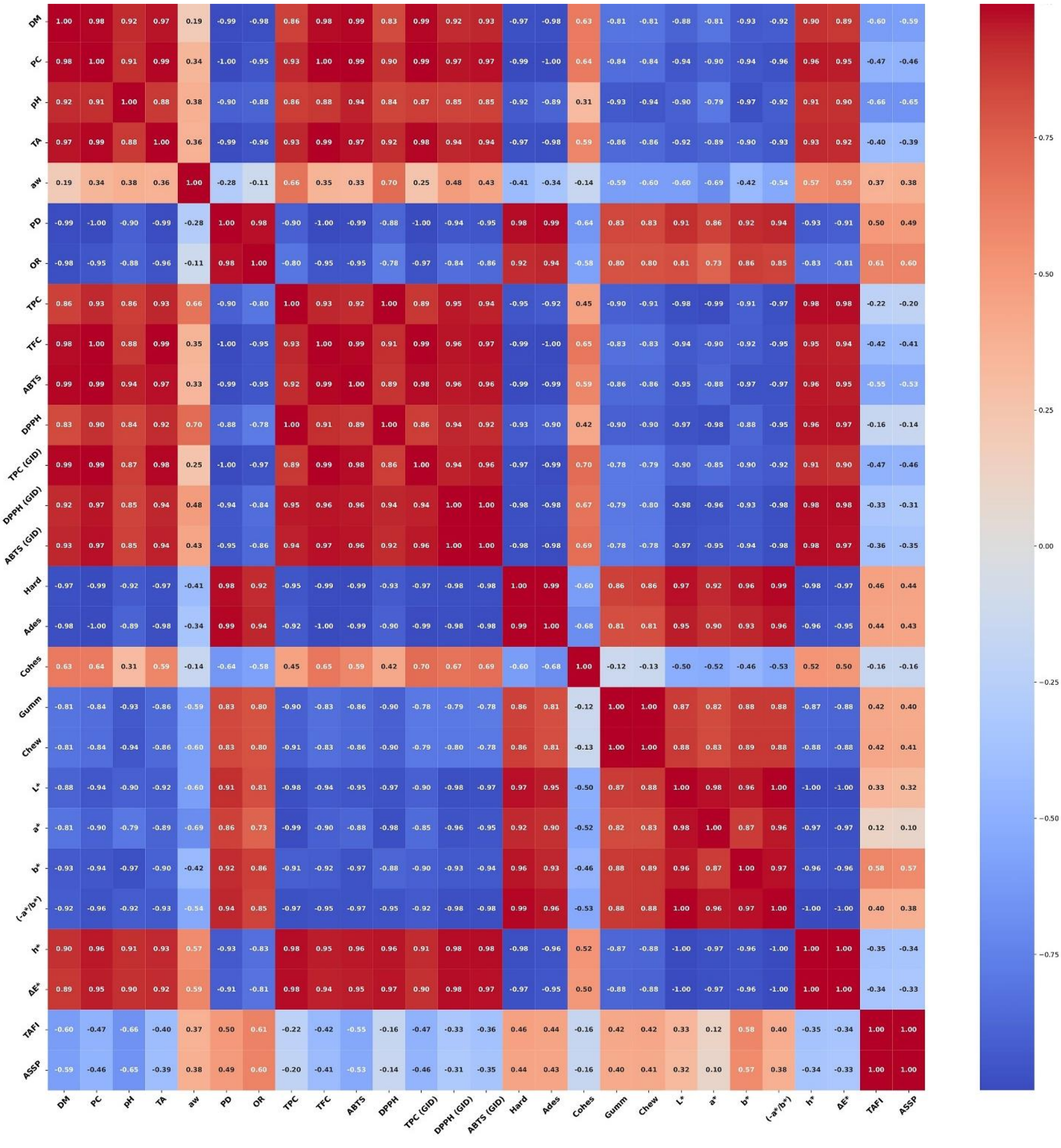

Figure S1. Feature correlation heatmap
